# Disruption of the eEF1A1/ARID3A/PKC‐δ Complex by Neferine Inhibits Macrophage Glycolytic Reprogramming in Atherosclerosis

**DOI:** 10.1002/advs.202416158

**Published:** 2025-02-20

**Authors:** Baoping Xie, Li‐Wen Tian, Chenxu Liu, Jiahua Li, Xiaoyu Tian, Rong Zhang, Fan Zhang, Zhongqiu Liu, Yuanyuan Cheng

**Affiliations:** ^1^ State Key Laboratory of Traditional Chinese Medicine Syndrome Key Laboratory for Translational Cancer Research of Chinese Medicine Joint Laboratory for Translational Cancer Research of Chinese Medicine of the Ministry of Education of the People's Republic of China International Institute for Translational Chinese Medicine School of Pharmaceutical Sciences Guangzhou University of Chinese Medicine Guangzhou Guangdong 510006 China; ^2^ Key Laboratory of Prevention and Treatment of Cardiovascular and Cerebrovascular Diseases (Gannan Medical University), Ministry of Education, Jiangxi Provincial Key Laboratory of Tissue Engineering Gannan Medical University Ganzhou Jiangxi 341000 China; ^3^ School of Pharmaceutical Sciences Southern Medical University Guangzhou 510515 China; ^4^ State Key Laboratory of Quality Research in Chinese Medicine Macau University of Science and Technology Macau 999078 China

**Keywords:** atherosclerosis, eEF1A1/ARID3A/PKC‐δ complex, macrophage glycolytic reprogramming, neferine

## Abstract

Glycolytic reprogramming of macrophages is a decisive factor in atherosclerosis (AS) plaque formation. Eukaryotic elongation factor 1A1 (eEF1A1) plays an important role in protein synthesis, ubiquitination degradation, and nuclear translocation. However, the potential function of eEF1A1 in AS has not yet been fully understood. Here, the natural small molecule neferine (Nef), which targets eEF1A1 to suppress macrophage glycolytic reprogramming is discovered. In this work, chemical genetics and non‐modified target confirmation assays are used to confirm that eEF1A1 is a direct target of Nef. Mechanically, Nef disrupted the formation of the eEF1A1/ARID3A/PKC‐δ complex, inhibits phosphorylation of ARID3A at Thr491, and consequently prevents its nuclear translocation. Meanwhile, it is verified that ARID3A is a transcriptional regulator of enolase 2 (ENO2), an important enzyme in the glycolytic process. Nef suppresses ENO2 transcription activation by affecting ARID3A binding to the promoter region of ENO2, which results in macrophage glycolytic reprogramming inhibition and transformation of macrophages from M1 to M2. Collectively, these findings provide an attractive future direction for AS therapy by inhibiting ARID3A/ENO2‐mediated macrophage glycolytic reprogramming by targeting eEF1A1.

## Graphical abstract

The eEF1A1/ARID3A/PKC‐δ complex critically regulates macrophage glycolytic reprogramming in atherosclerosis by mediating ARID3A phosphorylation and nuclear translocation, thereby driving ENO2 transcriptional activation. Neferine targets eEF1A1 to disrupt the eEF1A1/ARID3A/PKC‐δ complex to inhibit ENO2‐mediated macrophage glycolytic reprogramming in atherosclerosis.

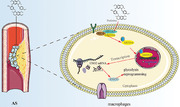

## 1 Introduction

Atherosclerosis (AS) serves as the primary pathological foundation for multiple cardiovascular diseases, such as myocardial infarction, angina pectoris, heart failure, and stroke.^[^ ^1^, ^2^, ^3^ ^]^ The pathological mechanism of AS is intricate and involves endothelial cell injury, lipid deposition, aggregation and infiltration of inflammatory cells, release of inflammatory factors, aggregation and activation of platelets, formation of macrophage foam cells, and proliferation and migration of vascular smooth muscle cells (VSMCs).^[^ ^2^, ^4^, ^5^ ^]^ Recently, numerous studies have shown that metabolic reprogramming is widespread in AS plaques, including VSMCs switch from a “contractile” phenotype to an extremely proliferative “synthetic” phenotype,^[^ ^6^ ^]^ and M1 macrophages switch from M2 macrophages,^[^ ^7^ ^]^ especially glycolytic reprogramming of macrophages is a decisive factor in AS plaque formation.^[^ ^8^, ^9^ ^]^

Macrophages, derived from peripheral monocytes, are the primary immune cells in AS plaques.^[^ ^10^, ^11^ ^]^ Classically activated M1 macrophages sustain inflammation and induce glycolytic reprogramming, inflammatory mediators, ROS, and lactic acid production, while alternatively activated M2 macrophages are oxidative phosphorylation (OXPHOS) reprogramming and resolve inflammation by secreting anti‐inflammatory cytokines such as IL‐4 and IL‐10, and maintain plaque stability or tissue repair.^[^ ^7^, ^12^, ^13^, ^14^ ^]^ During the early stages of AS, macrophages undergo glycolytic metabolic reprogramming‐driven transformation into a pro‐inflammatory phenotype in response to oxidized low‐density lipoprotein (ox‐LDL) stimulation, which further exacerbates vascular endothelial inflammation and contributes to the progression of AS.^[^ ^15^, ^16^, ^17^ ^]^ Therefore, inhibiting the glycolytic reprogramming of macrophages is a new strategy for the clinical treatment of atherosclerosis.^[^ ^18^ ^]^

Eukaryotic elongation factor 1A1 (eEF1A1), as a translation elongation factor, can catalyze the GTP‐dependent binding of aminoacyl‐tRNA (aa‐tRNA) to the A‐site of ribosomes during the elongation phase of protein synthesis.^[^ ^19^ ^]^ More evidence exhibits that eEF1A1 has noncanonical functions in diverse cellular processes, including regulating protein ubiquitination degradation, nuclear translocation, and metabolic substrate preference.^[^ ^20^, ^21^ ^]^ Knockdown of eEF1A1 influences glycolysis by decreasing the expression and activity of hexokinase 2 (HK2) in HepG2 cells.^[^ ^22^ ^]^ As an inhibitor of eEF1A1, didemnin B decreased high‐fat diet‐induced liver and plasma triglycerides levels, improved oral glucose tolerance, and decreased THP‐1 macrophage IL‐1β secretion.^[^ ^23^ ^]^ However, the potential function and underlying mechanism of eEF1A1 in the metabolic reprogramming of macrophages in AS has not yet been fully understood.

In this work, we identified an eEF1A1‐targeting natural small molecule, neferine (Nef), with an obvious anti‐atherosclerotic effect in vitro and in vivo. Mechanism study reveals that eEF1A1 interacted with ARID3A and PKC‐δ, promoted phosphorylation of ARID3A at Thr491 and the nuclear translocation, ARID3A is confirmed as a transcriptional regulator of enolase 2 (ENO2), a key enzyme in the glycolytic process. Disrupting the eEF1A1/ARID3A/PKC‐δ complex by Nef blocked the nuclear translocation of ARID3A and ENO2 transcription activation, which results in macrophage glycolytic reprogramming inhibition and transformation of macrophages from M1 to M2. In summary, our study provides a new strategy for eEF1A1‐targeting therapeutics for AS by inhibiting macrophage glycolytic reprogramming. Moreover, our studies underline the importance of eEF1A1/ARID3A/PKC‐δ complex in macrophage glycolytic reprogramming.

## 2 Results

### 2.1 Nef Alleviated High‐Fat Diet‐Induced AS

**Figure 1:**
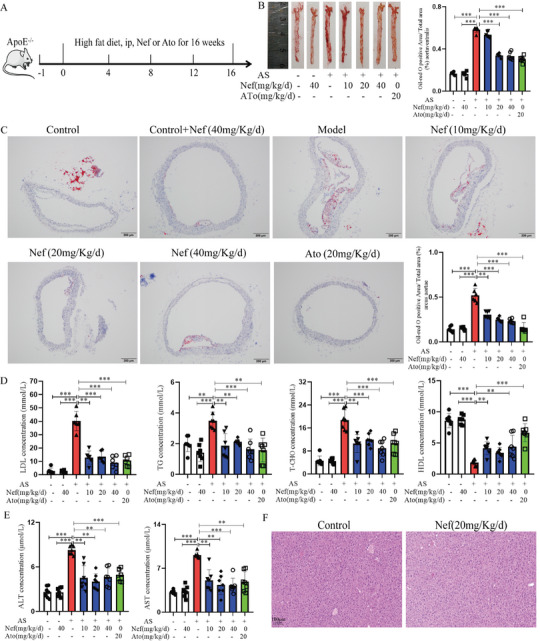
The pharmacological effects of Nef against atherosclerosis. A) In vivo experimental procedures for the antiatherogenic effect of Nef. B) Oil red staining and statistics of aortaventralis. C) Oil red staining and statistics of the aortic arch. D) Serum biochemical measurements of TC, T‐CHO, LDL, and HDL. E) Serum biochemical measurements of ALT and AST. F) H&E staining for liver tissue. ^*^ *p*< 0.05, ^**^ *p*< 0.01, ^***^ *p*< 0.001 versus Model group, scale column: 455100 µm, (*n* = 6).

To examine the anti‐atherosclerotic effect of Nef, ApoE^−/−^ mice were subjected to a high‐fat diet (HFD) for a duration of 16 weeks (**Figure** **1**A). The effectiveness of the model was assessed by quantifying the serum levels of T‐CHO, TG, LDL, and HDL. Additionally, AS preferentially develops in curved and branching arterial regions.^[^ ^24^ ^]^ Thus, the arcus aortae and aortaventralis were excised for oil‐red staining to determine the size of the AS plaques. The results showed that the Model group showed larger atherosclerotic lesion areas both in the aortaventralis and arcus aortae regions compared with the control mice. Interestingly, the atherosclerotic lesion areas were decreased after Nef at all concentrations and atorvastatin (Ato, 20 mg Kg^−1^ d^−1^) treatment, and the efficacy of Nef (20 and 40 mg Kg^−1^ d^−1^) was similar to that of Ato (Figure 1B,C). In terms of blood lipid regulation, the results showed that the levels of T‐CHO, TG, and LDL were significantly increased and the content of HDL was remarkably decreased in the Model group compared to the Control group. Nef at all concentrations treatment remarkably reduced the T‐CHO, TG, and LDL levels and enhanced the HDL level in serum (Figure 1D). The results of this study were consistent with the previous reports that Nef significantly reduced the Western‐style diet‐induced elevation of lipid levels and decreased the risk of non‐alcoholic fatty liver disease (NAFLD). Moreover, HFD‐induced significantly increased serum AST and ALT levels, which were significantly inhibited by different concentrations of Nef and atorvastatin compared with the Model group (Figure 1E). Furthermore, HE staining was used to examine the effect of Nef on liver function, and the results showed that the hepatocytes in the Control group and Nef group were normally arranged without inflammatory infiltration (Figure 1F). Therefore, our results present in vivo evidence demonstrating the favorable antiatherogenic effect of Nef, and Nef exhibited safety and efficacy at the experimental dosage.

### 2.2 Nef Inhibited Ox‐LDL‐Induced M1 Macrophage Polarization and Glycolytic Reprogramming

**Figure 2:**
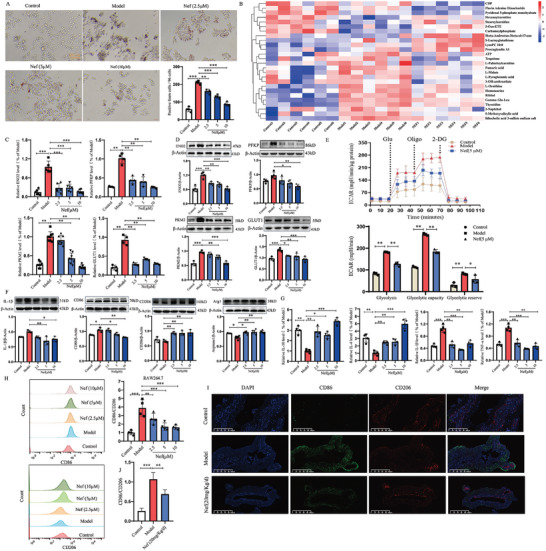
Effects of Nef on macrophage polarization and glycolytic reprogramming in ox‐LDL‐induced macrophages. A) Nef inhibited ox‐LDL‐induced macrophage foam cell formation in a dose‐dependent manner (*n* = 4). B) Quasi‐Targeted metabolomics analysis was used to evaluate the effect of Nef on macrophage metabolism (*n* = 6). C) The effect of Nef on the expression of differential genes induced by Ox‐LDL in macrophages (*n* = 3). D) The effects of Nef on glycolysis‐related gene expression. E) Nef inhibited ox‐LDL‐induced glycolysis levels and fluxes (*n* = 4). F) Nef inhibited ox‐LDL‐induced M1 macrophage polarization, and promoted the expression of M2 macrophage biomarkers (*n* = 4). G) Effect of Nef on the expression of IL‐10, IL‐4, IL‐1β and TNF‐α. H) Flow cytometry analysis for the ratio of CD86/CD206. I,J) Immunofluorescence images for CD86 and CD206 in AS plaques (*n* = 3). ^*^ *p*< 0.05, ^**^ *p*< 0.01, ^***^ *p*< 0.001, versus Model group, scale column:100 or 200 µm.

Firstly, we examined the toxicity of Nef on macrophage and found that Nef has no toxicity at experimental doses (Figure S1A, Supporting Information). Macrophage foaming usually occurs in the early stage of atherosclerosis. Peripheral monocytes pass through the vascular smooth muscle to the intima layer and transform into macrophages, macrophages transform into foaming macrophages under the stimulation of ox‐LDL.^[^ ^13^, ^18^ ^]^ The results in **Figure** **2**A showed that Nef dose‐dependently inhibited ox‐LDL‐induced macrophage foam formation. Generally, foam macrophage is considered as a pro‐inflammation macrophage, and their metabolism is mainly dominated by glycolysis and pentose phosphate pathway (PPP) induced by ox‐LDL.^[^ ^25^, ^26^ ^]^ Thus, we carried out quasi‐targeted metabolomics discovered that ox‐LDL significantly upregulated the levels of glycolysis‐related metabolites and promoted the accumulation of tricarboxylic acid cycle (TCA) metabolites, but Nef significantly inhibited the accumulation of TCA metabolites and promotes ATP synthesis (Figure 2B), especially fumaric acid and L‐ malate (Figure S1B,C, Supporting Information). KEGG analysis also showed that Nef regulated pyruvate metabolism and the OXPHOS pathway (Figure S1D, Supporting Information). Furthermore, we used RNA‐seq to investigate the differential gene expression regulated by Nef in ox‐LDL‐induced macrophages, and enrichment analysis showed that Nef significantly inhibited the expression of glycolysis‐related genes induced by ox‐LDL (Figure S1E, Supporting Information). Importantly, Nef dose‐dependently suppressed glycolysis‐related gene expression (ENO2, PKM2, PFKP, and GULT1) at the mRNA and protein level (Figure 2C,D; Figure S2A, Supporting Information). In addition, the seahorse experiments showed similar results that ox‐LDL significantly increased ECAR levels, glycolytic rate, and flux, and inhibited cellular oxygen consumption, while Nef significantly reduced ECAR and increased the OCR value and OXPHOS levels (Figure 2E, Figure S1F, Supporting Information). Meanwhile, we found that ox‐LDL stimulation significantly increased lactate, ROS, and MDA levels, but Nef notably inhibited these levels (Figure S1G–I, Supporting Information). Glycolysis is a typical feature of M1 macrophage metabolism.^[^ ^27^ ^]^ As shown in Figure 2F,G, Nef significantly inhibited the levels of M1 macrophage biomarkers (IL‐1β, TNF‐α, CD86) and up‐regulated the expression of M2 macrophage biomarkers (IL‐10, IL‐4, CD206, and Arg1). The flow cytometry results also indicated that Nef could significantly inhibit the ratio of C86/CD206 (Figure 2H; Figure S2B, Supporting Information). More importantly, we examined the effect of Nef on the expression of CD86 and CD206 in AS plaques by immunofluorescence, and the results showed that Nef inhibited the expression of CD86 and upregulated the expression of CD206 in abdominal aortic plaques, suggesting that Nef played a therapeutic role in AS by regulating the M1/M2 ratio (Figure 2I,J). Then, we also investigated the effect of Nef on the subtypes of M2 macrophages and found that Nef might be involved in ox‐LDL‐induced conversion of macrophages to M2a phenotype (Figure S1J–M, Supporting Information). Taken together, these results indicate that Nef inhibited ox‐LDL‐induced M1 macrophage polarization and glycolytic reprogramming.

### 2.3 eEF1A1 was Selectively Targeted by Nef

**Figure 3:**
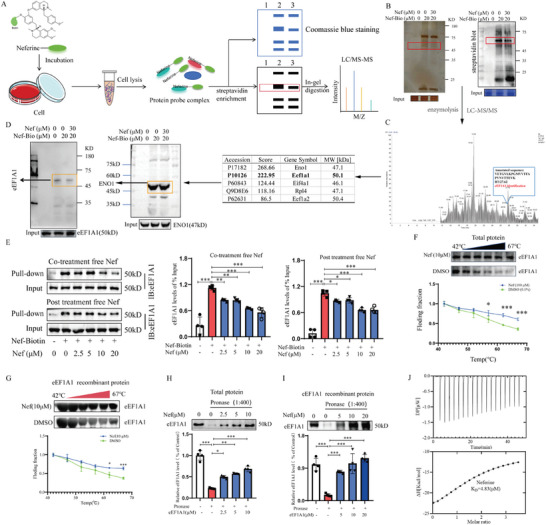
eEF1A1 was selectively targeted by Nef. A) Schematic representation of biotin‐coupled small molecule compound for target fishing. B) Silver staining and HRP‐ Streptavidin antibody assay for competitive antagonism. C) Direct targets of Nef action were identified by LC‐MS/MS. D) The specific antibody against eEF1A1 identified eEF1A1 as a potential direct target of Nef. E) Streptavidin magnetic bead pull down assay and statistical graph. F,G) CETSA using RAW264.7 macrophage intact cells and eEF1A1recombinant protein, which were exposed to Nef (10 µm) (*n* = 3). H,I) Nef promoted target protein eEF1A1 resistant to proteases (DARTS) in total protein and eEF1A1 recombinant protein (*n* = 4). J) ITC assay confirmed that eEF1A1 is a direct target of Nef. ^*^ *p*< 0.05, ^**^ *p*< 0.01, ^***^ *p*< 0.001, versus Model group or Control group.

To identify the direct target of Nef for inhibiting the glycolytic reprogramming of macrophages, a biotin‐tagged Nef probe (Nef‐Bio) was designed and synthesized (Figure S3A, Supporting Information). Then, we examined the cytotoxicity and pharmacodynamics of Nef‐Bio and found that Nef‐Bio (5 µm) significantly inhibited macrophage foam formation without cytotoxicity, and inhibited the expression of M1 biomarkers (IL‐1β, CD86) and promoted the expression of M2 biomarkers (CD206 and Arg1), which showed similar cellular potency to the unmodified Nef (5 µM) (Figure S3B–D, Supporting Information). Following pulldown onto streptavidin magnetic beads, biotinylated proteins labeled by Nef were separated by gel electrophoresis, visualized by coomassie blue dye or silver staining, and identified by LC‐MS/MS (**Figure** **3**A). As presented in Figure 3B, one differential protein band could be observed between ≈50 kDa in the pull‐down group with Nef beads; however, a much weaker protein band was found in the group with an excess amount of Nef for competition. LC/MS/MS analysis identified eEF1A1 as the major protein band at ≈50 kD (Figure 3C). Then, the identity of the biotin‐labeled protein was also confirmed by Western blotting with an anti‐eEF1A1 antibody and anti‐ENO1 antibody (Figure 3D). Interestingly, unmodified Nef competitively reduced Nef‐Bio bound eEF1A1 but had no effect on ENO1 abundance, suggesting that eEF1A1 is the major binding target for Nef rather than ENO1. More importantly, we performed a pull‐down experiment to verify the direct interactions between Nef and eEF1A1 (Figure 3E). Small‐molecule inhibitors can perturb protein function and increase the protein stability via forming a ligand–protein complex. Thus, we attempted to investigate whether Nef could bind to eEF1A1 protein and increase its stability in intact cells or lysate using cellular thermal shift assay (CETSA) and drug affinity responsive target stability (DARTs)assays (Figure 3F–I). CETSA results showed that Nef administration protected the eEF1A1 protein from temperature‐dependent degradation in cell lysis and eEF1A1 recombinant protein (Figure 3F,G). DARTs assay also confirmed that Nef induced stabilization of eEF1A1 protein, as well as eEF1A1 recombinant protein (Figure 3H,I). Furthermore, the results of ITC assay confirmed the affinity of Nef to eEF1A1 with the affinity constant of K_D_ = 4.83 µm, thermodynamic constants ΔH = −15.6 (kcal mol^−1^), ΔG = −7.25 (kcal mol^−1^) (Figure 3J). Collectively, these data suggest that eEF1A1 might be a direct target for Nef.

### 2.4 Nef Inhibited Ox‐LDL‐Induced Macrophage Glycolytic Reprogramming by Targeting eEF1A1

**Figure 4:**
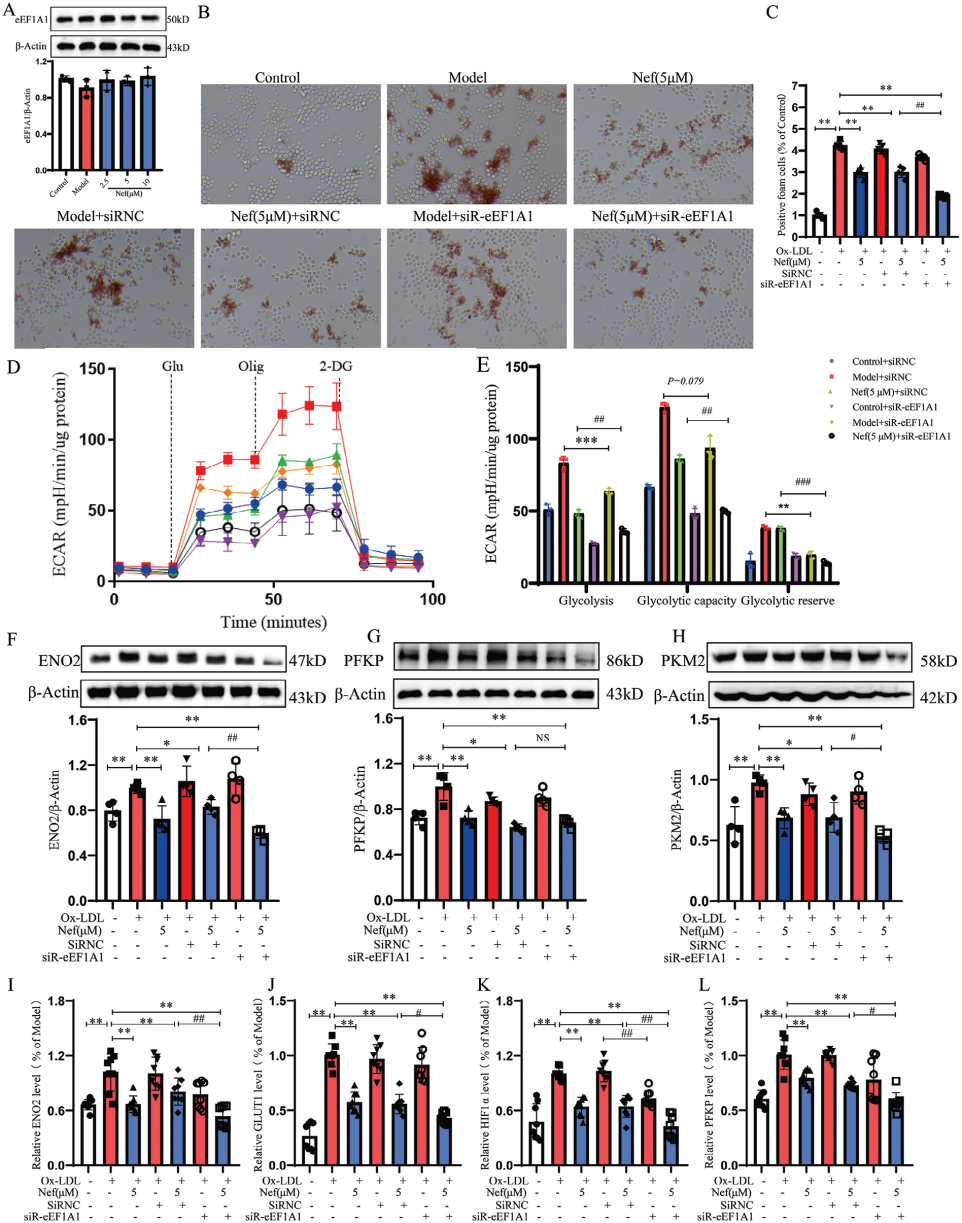
Nef inhibited ox‐LDL‐induced glycolysis reprogramming by targeting eEF1A1. A) Effect of Nef on eEF1A1 expression (*n* = 3). B,C) Effect of eEF1A1 silencing on the regulation of macrophage foam cell formation by Nef (*n* = 3). D,E) The effect of eEF1A1 silencing on the regulation of ox‐LDL‐induced macrophage glycolysis by Nef (*n* = 3). F–H) The effect of Nef on the protein expression of glycolysis‐related genes after silencing eEF1A1 (*n* = 4). I–L) The effect of Nef on the mRNA level of glycolysis‐related genes after silencing eEF1A1 (*n* = 5). ^*^ *p*< 0.05, ^**^ *p*< 0.01, versus Model group. ^#^ *p*< 0.05, ^##^ *p*< 0.01, versus Model + siRNC group or Nef (5 µm) +siRNC group, scale column:100 µm.

To further clarify the target role of eEF1A in regulating glycolytic reprogramming and phenotypic switching by Nef, we first examine the effect of Nef on the expression of eEF1A1, however, we found that Nef did not have a direct regulatory effect on the expression of eEF1A1 (**Figure** **4**A). Importantly, eEFE1A is highly expressed in macrophages, so we chose to use eEF1A1 siRNA to silence the eEF1A1 function (Figure S4A, Supporting Information). Surprisingly, when eEF1A1 was silenced, the effect of ox‐LDL on macrophage foam formation was slightly weakened (*p* > 0.05), but the inhibitory effect of Nef on macrophage foam formation was significantly enhanced (Figure 4B,C). Furthermore, the seahorse assay showed that silencing eEF1A1 significantly inhibited ox‐LDL‐induced glycolysis, promoted the maximum mitochondrial respiration capacity, and enhanced the inhibitory effect of Nef on glycolysis, and the regulation of OXPHOS (Figure 4D,E; Figure S4B,C, Supporting Information). Then, we examined the effect of silencing eEF1A1 on the expression of genes involved in glycolysis by Western Blot and qRT‐PCR. EEF1A1 knockdown inhibited the expression of glycolytic gene ENO2, PKM2, PFKP, GLUT1, and HIF1α at the mRNA and protein level (*p* < 0.05), and enhanced the inhibitory effect of Nef on the expression of these genes, especially ENO2 expression (*p* < 0.01). The results suggest that Nef inhibits ox‐LDL‐induced glycolytic reprogramming by targeting eEF1A1 (Figure 4F–L). In addition, we also found that silencing eEF1A1 suppressed ox‐LDL‐induced M1 macrophage polarization and the inhibitory effect of Nef on M1 macrophage polarization was enhanced (Figure S4D, E, H, I, Supporting Information). Meanwhile, eEF1A1 knockdown promoted the increased effect of Nef on M2 cytokine secretion and biomarker expression (IL‐4、IL‐10、CD206 and Arg1) (Figure S4F, G, J‐M, Supporting Information). Thus, eEF1A1 might be a direct target of Nef for suppressing ox‐LDL‐induced glycolysis reprogramming and M1 macrophage polarization.

### 2.5 ARID3A Severed as a Key Transcription Factor for ENO2 Regulated by Nef

**Figure 5:**
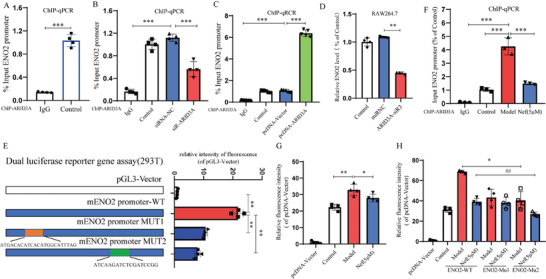
ARID3A is a transcription factor of ENO2. A) The effect of ARID3A on the ENO2 transcription activation measured by ChIP‐qPCR (*n* = 4). B,C) ARID3A silencing and overexpression on the ENO2 transcription activation detected by ChIP‐qPCR (*n* = 4). D) The effect of ARID3A on ENO2 mRNA level. E) Dual luciferase‐reporter assays of ENO2 transcriptional activity in 293T cells. F,G) The effect of Nef on the transcriptional activity of ENO2 detected by luciferase reporter assay and ChIP assay. H) Dual luciferase reporter assay confirmed that ATCAAGATCTCGATCCGG was the key site of Nef for regulating the ENO2 transcriptional activity. ^*^ *p*< 0.05, ^**^ *p*< 0.01, versus Model group, ^#^ *p*< 0.05, ^##^ *p*< 0.01, versus Nef (5 µm) group.

Considering that Nef inhibited ENO2 transcription (Figure 2C; Figure S1E, Supporting Information), an important kinase in glycolysis. Then, we used the JASPAR database (https://jaspar.elixir.no/) to predict that ARID3A may be a transcriptional regulator of ENO2, and identified by ChIP assay (**Figure** **5**A). To further confirm that ARID3A is a transcription factor for ENO2, we screened siRNA‐ARID3A to silence the ARID3A function (Figure S5A, Supporting Information). Then, the selected siR‐ARID3A and pcDNA‐ARID3A were transfected into RAW264.7 cells, and ChIP assay confirmed that ARID3A silence reduced the chromatin pull down of ENO2, while ARID3A overexpression significantly increased the amount of ENO2 (Figure 5B,C). Meanwhile, silencing ARID3A significantly inhibited the mRNA expression of ENO2 in RAW264.7 and 293T cells (Figure 5D; Figure S5B, Supporting Information). All the results indicated that ARID3A is a transcription factor of ENO2. Importantly, we also confirmed the key sequence of ARID3A for regulating ENO2 transcription by dual luciferase reporter gene assay. ARID3A has two binding sites with ENO2: “ATGACACATCACATGGCATTTAG” and “ATCAAGATCTCGATCCGG”(Figure 5E). More importantly, we examined the effects of Nef and ox‐LDL on the regulation of the ARID3A/ENO2 pathway by ChIP and dual luciferase reporter gene experiments. Interestingly, the results showed that ox‐LDL significantly promoted the transcriptional activity of ARID3A on ENO2 and compared with the Control group, but compared Model group, Nef inhibited the regulation of ARID3A on ENO2 and significantly reduced relative fluorescence intensity (Figure 5F,G), Furthermore, we found that ENO2 mutation enhanced the inhibitory effect of Nef on ARID3A transcription of ENO2, especially pGL3‐Basic and mENO2 promoter MUT2, suggesting that “ATCAAGATCTCGATCCGG” was the key site of Nef targeted regulation of ARID3A‐mediated ENO2 transcription (Figure 5H). Taken together, ARID3A is a key transcriptional regulator of ENO2, and the transcription regulatory sites may be located in the “ATGACACATCACATGGCATTTAG” and “ATCAAGATCTCGATCCGG.” These results suggested that Nef may regulate glycolytic reprogramming by inhibiting ENO2 transcription through ARID3A/ENO2 pathway.

### 2.6 Nef Disrupted eEF1A1/ARID3A/PKC‐δ Complex to Promote the Phosphorylation and Nuclear Translocation of ARID3A

**Figure 6:**
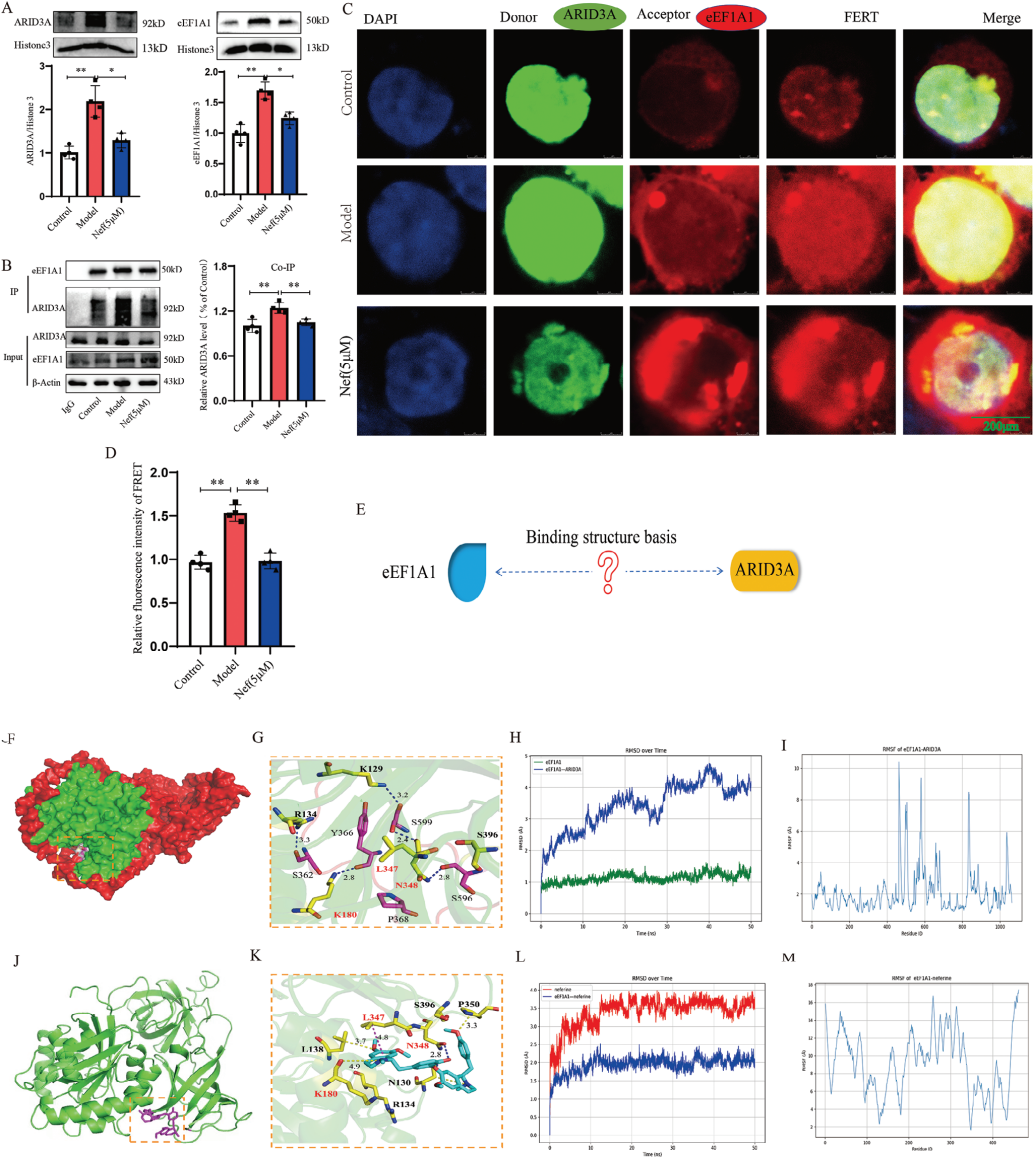
Nef interfered with the interaction between eEF1A1 and ARID3A. A) The effect of Nef on the nuclear translocation of ARID3A and eEF1A1. B) Nef suppressed the interaction between ARID3A and eEF1A1 by Cthat o‐IP assay. C,D) FRET assay confirmed that ARID3A interacted with eEF1Al. E,F) Protein surface diagram of the computational protein‐protein binding structure of eEF1A1‐ARID3A. The eEF1A1 and ARID3A are rendered in green and red respectively. G) Close up of the protein‐protein interaction interface of eEF1A1‐ARID3A. The eEF1A1 and ARID3A residues are rendered in yellow and magenta. Hydrogen bond interactions are labeled with blue dashed lines. H,I) RMSD and RMSF plot of eEF1A1‐ARID3A. J) The computational protein‐ligand binding structure of eEF1A1‐neferine complex. K) Close up of neferine binding site of eEF1A1. The eEF1A1 residues and ligand neferine are rendered in yellow and cyan. Hydrogen bond interaction, CH‐π interaction, and the distance between neferine and near residues are labeled with blue, magenta, and yellow dashed lines respectively. L,M) RMSD and RMSF plot of eEF1A1‐neferine.

To clarify the mechanism of Nef on inhibiting ARID3A/ENO2‐mediated glycolytic reprogramming, we first examined the effect of Nef on ARID3A expression. Interestingly, Nef did not affect the expression of ARID3A at both the mRNA and protein levels (Figure S5C,D, Supporting Information), but Nef inhibited the nuclear import of ARID3A and eEF1A1 (**Figure** **6**A). Considering the function of eEF1A1 for regulating nuclear translocation, we speculated that eEF1A1 might have interact with ARID3A. Thus, we performed fluorescence resonance energy transfer (FRET) and CoIP experiments, and found that ox‐LDL significantly promoted the interaction between eEF1A1 and ARID3A, but Nef inhibited the complex of eEF1A1 and ARID3A (Figure 6B–D). To understand the structure basis of eEF1A1/ARID3A complex (Figure 6E), we used computational simulations to analyze the protein‐protein interactions between eEF1A1 and ARID3A. The protein‐protein binding structures were determined using H‐dock. MD simulations were then performed to further refine the complex structure and examine the interactions (Figure S6, Supporting Information). As illustrated in Figure 6F,G, intermolecular hydrogen bond interactions exist between K129‐S599, K180‐Y366, R134‐S362, L347‐S599, and N348‐S596 (with front residue number corresponding to eEF1A1, and the back residue number corresponding to ARID3A). The RMSD and RMSF analyses (depicted in Figure 6H,I) of the eEF1A1‐ARID3A complex revealed that the eEF1A1 remains highly stable, with an RMSD value below 1.5 Å. In contrast, fluctuations are observed in ARID3A, particularly within its loop regions. Notably, these fluctuating regions do not reside at the binding interface, suggesting that they will not substantially impact the protein‐protein interactions. Moreover, we also analyzed the binding and interactions between Nef and eEF1A1 using molecular docking and MD simulations. Six binding poses were identified through pocket searching and ligand docking, as illustrated in Figure S7 (Supporting Information). The binding pocket with the most favorable predicted binding score was selected for subsequent MD simulations. As depicted in Figure 6J,K, the primary interactions between eEF1A1 and Nef consist of a hydrogen bond formed between N348 and a neferine hydroxy group, as well as a CH‐π interaction between L347 and the aromatic ring of Nef. Additional residues surrounding the binding site within a 5 Å radius include L138, K180, P350, and S396. The RMSD and RMSF analyses (presented in Figure 6L,M) of the eEF1A1‐neferine complex demonstrated minor fluctuations in both the neferine molecule and the binding site residues of eEF1A1. This indicates that Nef adopts a relatively stable binding conformation within the eEF1A1 binding site. Importantly, a comparison of the protein‐ligand (eEF1A1‐neferine) and protein‐protein (eEF1A1‐ARID3A) interactions revealed that the binding sites of Nef on eEF1A1 spatially overlap with the binding interface of eEF1A1‐ARID3A. In other words, the binding of Nef directly occupies the space required for protein‐protein interactions, disrupting the formation of crucial interactions between key residues (K180, L347, and N348) of eEF1A1 and ARID3A. These findings suggest a competitive inhibitory effect of Nef toward the eEF1A1‐involved protein‐protein interaction.

**Figure 7:**
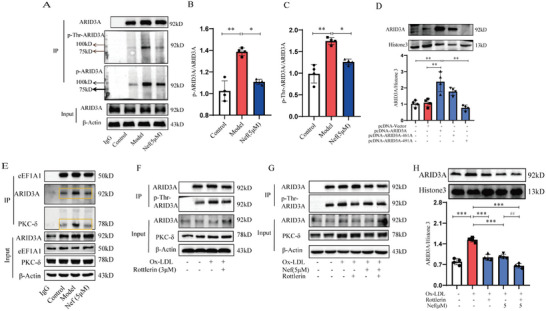
Nef disrupted eEF1A1/ARID3APKC‐δ complex to inhibit the phosphorylation and nuclear translocation of ARID3A. A–C) Nef inhibited ARID3A phosphorylation in particular threonine phosphorylation. D) Phosphorylation of ARID3A threonine at position 491 was a key site for ARID3A nuclear translocation. E) Nef inhibited the formation of eEF1A1/ARID3A/PKC‐δ complex. F) PKC‐δ was required for ARID3A phosphorylation. G) Nef inhibited the phosphorylation of ARID3A through PKC‐δ. H) Nef inhibited the nuclear translocation of ARID3A through PKC‐δ. ^*^ *p*< 0.05, ^**^ *p*< 0.01, versus Model group or rottlerin group, (*n* ≥ 3).

**Figure 8:**
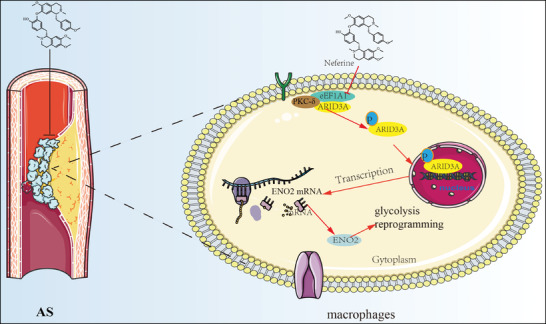
Mechanism of Nef on inhibiting macrophage glycolytic reprogramming induced by ox‐LDL.

It is reported that phosphorylation favors the transcription factor into the nucleus,^[^ ^28^, ^29^ ^]^ thus we evaluated the phosphorylation level of ARID3A. The results showed that ox‐LDL significantly increased the expression of p‐ARID3A, especially the level of threonine phosphorylation of ARID3A, and Nef significantly inhibited the expression of p‐ARID3A and p‐Thr‐ARID3A (**Figure** **7**A–C). Furthermore, we found that the domain that regulates ARID3A nuclear import may be located between 450 and 493 by UniProt analysis (https://www.uniprot.org/uniprotkb/Q62431/entry) (Figure S5E, Supporting Information), and there are only two threonine sites at 461 and 491. Therefore, we mutated the threonine at 461 and 491 to alanine and detected the nuclear translocation and phosphorylation of ARID3A by western blot. We found that mutation of THR at 491 significantly reduced ARID3A nuclear import and p‐Thr‐ARID3A expression, suggesting that phosphorylation of THR at 491 is a key site for ARID3A nuclear import (Figure 7D). Previous studies have shown that eEF1A1 can interact with PKC‐δ, and suggest that ARID3A phosphorylation may be related to PKC‐δ recruitment by eEF1A1. Interestingly, we found that ox‐LDL significantly promoted the formation of eEF1A1/p‐Thr‐ARID3A/PKC‐δ complex, while Nef inhibited the formation of the complex. Importantly, a PKC‐δ specific inhibitor (Rottlerin) significantly reduced ox‐LDL‐induced p‐Thr‐ARID3A expression and nuclear translocation and enhanced the inhibitory effect of Nef on ARID3A nuclear import and phosphorylation. These results suggest that the binding of Nef to eEF1A1 can inhibit the recruitment of PKC‐δ, which in turn leads to a decrease in the phosphorylation of ARID3A (Figure 7E–H). Taken together, Nef suppressed the glycolytic reprogramming of macrophages by interfering with the formation of eEF1A1/ARID3A/PKC‐δ complex, subsequently inhibiting the phosphorylation at THR491 and the nuclear translocation of ARID3A, thereby inhibiting ENO2 transcription activation (**Figure** **8**).

## 3 Discussion

Glycolytic reprogramming of macrophages is a decisive factor inAS plaque formation.^[^ ^30^, ^31^ ^]^ In the early stage of AS, ox‐LDL stimulated macrophage glycolysis significantly enhanced, subsequently glycolytic products ROS and lactate promote macrophage phenotype switching to M1 macrophages.^[^ ^12^ ^]^ At the same time, inflammatory factors released by M1 macrophages further aggravate vascular inflammation and endothelial injury, thereby accelerating the formation of AS plaque.^[^ ^32^, ^33^ ^]^ Thus, inhibiting the glycolytic reprogramming of macrophages may be a new strategy for anti‐AS. In this work, we discovered a novel mechanism for ox‐LDL‐induced macrophage glycolytic reprogramming in AS. Specifically, eEF1A1 formed the complex with ARID3A and PKC‐δ, promoting phosphorylation and the nuclear translocation of ARID3A at THR491, subsequently activating ENO2 transcription to enhance macrophage glycolytic reprogramming. Nef, a natural small molecule derived from *Plumula nelumbinis*, can disrupt the eEF1A1/ARID3A/PKC‐δ complex to suppress ARID3A nuclear translocation and ENO2 transcription activation. This disruption ultimately inhibits glycolytic reprogramming in macrophages and reduces the formation of AS plaques.

eEF1A1, as an essential target in protein elongation,^[^ ^34^ ^]^ has extensive involvement in both protein synthesis and ubiquitination degradation^[^ ^21^, ^34^ ^]^ as well as mediation of nuclear translocation.^[^ ^20^ ^]^ Additionally, eEF1A1 significantly contributes to cell proliferation and migration. Inhibition of eEF1A1 or reduction of p‐eEF1A1 expression in microglia promoted its polarization to M1 phenotype.^[^ ^35^, ^36^, ^37^, ^38^ ^]^ Moreover, silencing the expression of eEF1A1 reduced the expression of HK2, thereby reducing the glycolysis level of HepG2 cells.^[^ ^22^ ^]^ Taken together, these studies point to the important role of eEF1A1 in cell metabolism and polarization. Similar to these studies, our study also found that the silencing of eEF1A1 significantly promoted the oxygen consumption of macrophages and reduced their glycolysis levels. This finding further confirms the key role of eEF1A1 in regulating macrophage metabolism and provides strong evidence for its role as a key target for the glycolytic reprogramming of macrophages. Importantly, we used a biotin‐labeled small probe (Nef‐Bio), LC/MS/MS, CETSA, and DARTS assays to identify and confirm eEF1A1 as the key direct target of Nef. Moreover, eEF1A1 silence  significantly enhanced the inhibitory effect of Nef on macrophage glycolysis by featured as inhibiting the mRNA and protein expression of glycolysis‐related genes (ENO2, PFKP, GLUT1, and HIF1α), and promoted M2 macrophage polarization.

ENO2 is one of the key rate‐limiting enzymes of glycolysis, which can regulate the production rate of phosphoenolpyruvate, phosphoenolpyruvate induces endothelial dysfunction and cell senescence through stimulation of metabolic reprogramming.^[^ ^39^ ^]^ In our study, we found that the level of glycolysis decreased while the level of OXPHOS increased when ENO2 was reduced. The underlying mechanisms for this may involve adjustment of glycolysis and oxidative phosphorylation balance. To meet the energy needs of cells, the pyruvate produced by glycolysis is transported to the mitochondria, where it is converted to acetyl‐CoA by the pyruvate dehydrogenase complex, entering the tricarboxylic acid cycle, providing more reducing equivalent (NADH and FADH) for oxidative phosphorylation, facilitating the oxidative phosphorylation process.^[^ ^40^, ^41^ ^]^ Interestingly, our RNA‐seq and qRT‐PCR data showed that Nef significantly inhibited the expression of ENO2 at mRNA level in ox‐LDL‐induced macrophages. Considering the inhibitory effect of Nef on ENO2 transcription, we predicted by JASPAR database and confirmed ARID3A as a key transcription factor for ENO2 by ChIP and dual‐luciferase reporter assays. ARID3A belongs to a large family of A+T rich interaction domain proteins and acts as a transcription factor regulating gene expression.^[^ ^42^ ^]^ ARID3A was originally reported to be specifically upregulated in activated B cells, promoting immunoglobulin transcription, and ARID3A overexpression promotes granulocytic/monocytic differentiation in murine fetal liver cells or human peripheral blood CD34^+^ cells.^[^ ^43^ ^]^ Moreover, ARID3A can regulate the expression of glycolysis‐related genes, including HIF1α, PDH, PFKFB3, GAPDH, SDH, and IDH.^[^ ^44^ ^]^ Unfortunately, these results are based on database predictions without laboratory confirmation, but we have experimentally confirmed that ARID3A is a transcription factor of ENO2. More importantly, we found that the sequences of “ATGACACATCACATGGCATTTAG” and “ATCAAGATCTCGATCCGG” were the key the binding sites for ARID3A on the promoter region of ENO2. Mutation of the sites, and the transcription activation of Nef on ENO2 were also inhibited.

Moreover, eEF1A1 and ARID3A were not significantly changed in ox‐LDL‐induced macrophages, however, the nuclear translocation of them was increased. eEF1A1 recruits PKC‐δ to promote STAT3 phosphorylation into the nucleus,^[^ ^45^ ^]^ and eEF1A1 rapidly activates transcription of HSP70 by recruiting the master regulator HSF1 to its promoter in response stress,^[^ ^46^ ^]^ which revealed the important role of eEF1A1 in protein nuclear translocation by binding with the relative protein. Thus, we used FERTs and CoIP assays to discover that eEF1A1 directly interacted with ARID3A, and the interaction were enhanced by ox‐LDL. More importantly, previous researches have shown that eEF1A1 can recruit PKC‐δ, PASK, and PKC‐α to promote the phosphorylation level of downstream proteins.^[^ ^38^, ^45^ ^]^ In our study, we also found that phosphorylation of ARID3A at THR491 was increased which was a key site for ARID3A nuclear import. eEF1A1、ARID3A and PKC‐δ could form the complex induced by ox‐LDL, and using the specific inhibitor of PKC‐δ, rottlerin, inhibited ARID3A phosphorylation and nuclear translocation. Compared with previous studies, our study not only explored the regulatory mechanism of eEF1A1 in depth but also revealed for the first time the important role of eEF1A1/ARID3A/PKC‐δ complex in the glycolytic reprogramming of macrophages. Interfere with protein‐protein interactions are the key target for several diseases and drug screening,^[^ ^47^, ^48^ ^]^ such as NCOA4/FTH1 complex often as a new mechanism for screening of ferroptosis inhibitor compounds.^[^ ^49^ ^]^ In the present study, Nef had no effect on the expression of eEF1A1 and ARID3A, however, Nef significantly inhibited the interaction between eEF1A1 and ARID3A, as well as ARID3A nuclear import and threonine phosphorylation. Moreover, the inhibitory effect of Nef on ARID3A nuclear import and threonine phosphorylation was enhanced by a PKC‐δ specific inhibitor. Nef also significantly inhibited the formation of eEF1A1/ARID3A/PKC‐δ complex. These results suggest that eEF1A1/ARID3A/PKC‐δ complex can be used as a target for high‐throughput screening of drugs for regulating the glycolytic reprogramming of macrophages.

However, our study still has some limitations: 1)We employed computational predictions to identify the key amino acid residues (L347, K180, N348) for eEF1A1/ARID3A complex formation, but still lacks experiment validation. We will construct the stably transfected cell line of eEF1A1‐knockout macrophages and transfected the eEF1A1 WT and mutations at the key amino acid residues (L347, K180, N348) virus plasmids to evaluate the drug efficacy of Nef. 2) Our study lacks the metabolism of Nef in vivo and the effects of its metabolites on atherosclerotic plaque activity and macrophage metabolic reprogramming. 3) Our study lacks detection of the impact on macrophage glycolytic reprogramming at different time points.

Collectively, our findings revealed eEF1A1 as a promising therapeutic target for AS by inhibiting macrophage glycolytic reprogramming. Moreover, we identified the formation of eEF1A1/ARID3A/PKC‐δ complex was important for macrophage glycolytic reprogramming by promoting ARID3A phosphorylation and nuclear import, leading to ENO2 transcription activation. Thus, disrupting the eEF1A1/ARID3A/PKC‐δ complex represents a valuable druggable target for designing lead compounds for AS therapy. Meanwhile, our study identified that Nef inhibited the formation of eEF1A1/ARID3A/ PKC‐δ complex to regulate the expression of ENO2 by targeting eEF1A1, thereby inhibiting ox‐LDL‐induced glycolytic reprogramming of macrophages.

## 4 Experimental Section

### Nef‐Bio Probe Synthesis


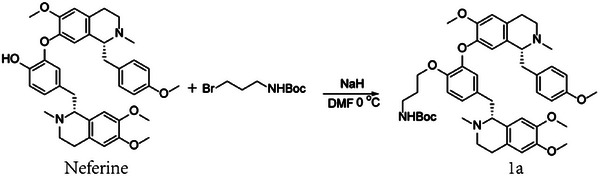


### Nef‐Bio Probe Synthesis

To a solution of neferine (100 mg, 0.16 mmol, Chengdu Herbpurify Co., Ltd., purity ≥ 98%) in anhydrous DMF (2 mL) was added sodium hydrate (0.24 mmol, 1.5 eq.) at 0 °C, and kept for 5 min. Then *tert*‐butyl (3‐bromopropyl) carbamate (0.32 mmol, 2 eq.) was added to the mixture and the reaction was stirred for 2 h. The reaction was quenched with H_2_O (10 mL), and extracted with ethyl acetate (50 mL) for three times. The combined ethyl acetate soluble portion was washed with brine and dried with anhydrous Na_2_SO_4_. The organic phase was concentrated and purified over silica gel (n‐hexane‐EtOAc, 1:1) to yield 1a (100 mg, yielding 95%). ^1^H NMR (400 MHz, CDCl_3_) δ_H_ 6.92 (d, *J* = 8.5 Hz, 2H), 6.82 (d, *J* = 8.2 Hz, 1H), 6.71 (m, 1H), 6.69 (d, *J* = 8.5 Hz, 2H), 6.66 (s, 1H), 6.65 (s, 1H), 6.52 (s, 1H), 6.30 (s, 1H), 6.08 (s, 1H), 4.01 (t, *J* = 5.9 Hz, 2H), 3.83 (s, 3H), 3.81 (s, 3H), 3.73 (s, 3H), 3.60 (s, 3H), 3.20‐3.07 (m, 7H), 3.00‐2.97 (m, 1H), 2.73‐2.86 (m, 7H), 2.49 (s, 3H), 2.46 (s, 3H), 1.87 (m, 2H), 1.38 (s, 9H). (+)‐ESIMS: 782.57 [M+H]^+^.


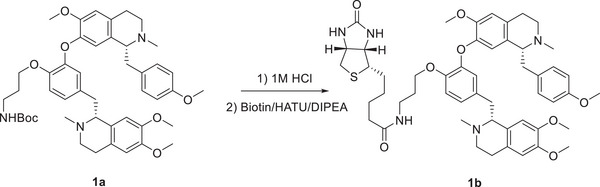


### Compound 1a

(100 mg, 0.13 mmol) in a round flask was added with 1 m HCl in 1,4‐dioxane (2 mL), and the solution was kept for 4 h at room temperature. Then the mixture was concentrated under reduced pressure and re‐dissolved in CHCl_3_ (5 mL). The CHCl_3_ solution was diluted with n‐hexane (100 mL), and concentrated to remove the excess amount of HCl. The yielded solid was re‐dissolved in DMF (2 mL), to which was added 2‐(7‐azabenzotriazol‐1‐yl)‐*N*,*N*,*N*″,*N*″‐ tetramethyluronium hexafluorophosphate (HATU, 0.25 mmol, 2 eq.), *N*,*N*‐diisopropylethylamine (0.52 mmol, 4 eq.), and D‐biotin (0.26 mmol, 2 eq.). 4 h later, the reaction was quenched with H_2_O (10 mL), and extracted with ethyl acetate (50 mL) for three times. The combined ethyl acetate soluble portion was washed with brine and dried by anhydrous Na_2_SO_4_. The organic phase was concentrated and purified over reversal phase silica gel (MeOH‐H_2_O, 1:1 – 1:0) to yield 1b (50 mg, yielding 42%). ^1^H NMR (400 MHz, CDCl_3_) δ_H_ 6.92 (d, *J* = 8.0 Hz, 2H), 6.83 (d, *J* = 8.3 Hz, 1H), 6.73 (m, 1H), 6.71 (m, 1H), 6.69 (d, *J* = 8.5 Hz, 2H), 6.57 (s, 1H), 6.53 (s, 1H), 6.28 (s, 1H), 6.08 (s, 1H), 4.01 (t, *J* = 5.9 Hz, 2H), 3.83 (s, 3H), 3.79 (s, 3H), 3.72 (s, 3H), 3.61 (s, 3H), 2.56 (s, 3H), 2.49 (s, 3H). (+)‐ESIMS: 908.72 [M+H]^+^.

### Cell Culture

RAW264.7 and 293T cells were purchased from the ATCC and Procell (Wuhan, China). These cells were cultured in Dulbecco's modified Eagle's medium (Gibco, USA) with 10% fetal bovine serum (Corning, New York, USA), 100 U mL^−1^ penicillin and streptomycin (Solarbio, China), and maintained at 37 °C with 5% CO_2_ humidified atmosphere. The cell was passaged regularly and grown to 70% confluence before treatments. Then, RAW264.7 cells were seeded in 96‐well plate at a density of 5 × 10^3^ cells/well for overnight, and then incubated with different concentrations of Nef or Nef‐Biotin (Nef‐Bio) for 24 h with or without ox‐LDL (100 ng mL^−1^) (Yuan ye, Shanghai, China). CCK‐8 kit (Biosharp, China) was used to measure the viability according to the manufacturer's instructions.

### Animal Experiments

A total of 8 male C57BL/6 wild type (WT) mice and 18 of ApoE^−/−^ mice on a C57BL/6 background (6‐ to 8‐week, 18–22 g) were purchased from the laboratory animal center of Southern Medical University (Guangzhou, China). This study was approved by the Ethics Committee of Guangzhou University of Traditional Chinese Medicine (20220313 and 20240203). The animal experiments were performed according to the Guide for the Care and Use of Laboratory Animals. After 1week adaptation, the ApoE^−/−^ mice were randomly divided into the model group, Nef (10, 20, and 40 mg Kg^−1^ d^−1^) groups, atorvastatin group with a high‐fat diet for a total of 16 weeks, and the normal‐chow‐fed wild‐type C57BL/6 mice were considered as control mice. Nef groups and atorvastatin (20 mg kg^−1^ d^−1^) group were gavaged with corresponding concentrations of the drugs, respectively. The mice in the Model group and Control group received an equal volume of normal saline. After 16‐week of treatment, the mice were sacrificed, and the aortaventralis, arcus aortae, livers, and serum were collected for further data analysis, such as hematoxylin‐eosin (H&E) staining, biochemical detection, and Oil Red O straining.

### Biochemical Assays

The levels of AST, ALT, LD, low‐density lipoprotein (LDL), high‐density lipoprotein (HDL), cholesterol (CHO), and triglyceride (TG) from serum or medium were measured using a biochemical test kit (Jiancheng Biotechnology, Nanjing, China) following manufacturer's instructions. The levels of ROS and MDA from RAW264.7 cells were measured using a biochemical test kit (Elabscience, Wuhan, China) following the manufacturer's instructions.

### Oil Red O Staining

The atherosclerotic lesions of mouse aortaventralis and arcus aortae and foam macrophages were assessed using Oil Red O staining. The sections were stained with oil‐red O (O0625, Sigma) for 5 min and then washed by PBS three times. Section images were captured by microscope. The rates of atherosclerotic lesion areas and positive foam macrophages were displayed as a percentage of oil‐red O positive area or numbers of red cells to the total staining area or total cells by Image J analysis software.

### Western Blotting

Total protein from RAW264.7 was extracted by using the RIPA lysis buffer (G2002‐100ML, Servicebio) containing 1 mm PMSF (P0100, Solarbio). The protein concentration of each sample was measured by Bradford (PC0020, Solarbio) and then the equal amount of protein was separated by 10% SDS‐PAGE and transferred onto a PVDF membrane. Then the membranes were blocked with 5% BSA for 1 h and incubated with the primary antibodies against CD86, IL‐1β, Arginase 1 (Arg 1), CD206, eEF1A1, ARID3A, ENO2, PKM2, ENO1 and β‐Actin (Cat. A20803, A16288, A1847, A11192, A23515, A7668, A12341, A1033 and AC038, Abclonal, Wuhan, China), or GLUT1, PFKP and PKC‐δ (ET1601‐10, HA500472, ET1701‐85, HUABIO, China) and HIF1α (BF8002, affinity, China) at 4 °C overnight. Then the membranes were incubated with a secondary antibody Goat Anti‐Rabbit IgG‐HRP (A0208, Beyotime, Beijing, China) for 1 h at 37 °C. The β‐Actin was used as an internal reference. The protein bands were visualized using ECL reagent (G3308, GBCBIO, China), and the optical densities of bands were detected using the image soft Gel‐Pro‐Analyzer.

### Quantitative Real‐Time Polymerase Chain Reaction (qRT‐PCR)

Total RNA was extracted by Trizol reagent (Qiagen, Frederick, MD, USA) according to the manufacturer's instructions. cDNA was synthesized by reverse transcription kit (AG11705, Accurate biology, Changsha, China) according to the manufacturer's instructions. The primers for TNF‐α, IL‐1β, IL‐4, IL‐10, and β‐Actin were purchased from Sangon Biotech (Shanghai, China) (Table S1, Supporting Information). qPCR was performed on a Real‐Time qPCR Thermal Cycler (Applied Biosystems, USA) using the SYBR Green method (AG11702, Accurate biology, Changsha, China). The data were analyzed by the ΔΔCt method, and β‐Actin was used as an internal control.

### Pull Down and LC‐MS/MS‐Based Targets Identification

Experimental procedures as previously described^[^ ^50^ ^]^ RAW264.7 cells were pre‐treated with competitors for 4 h, followed by adding the Nef‐Bio (20 µmol L^−1^) or DMSO in fresh medium. After incubation for 4 h, the soluble proteins were extracted to perform RIPA lysate. The protein concentration of each sample was measured by Bradford (PC0020, Solarbio, China). Then, the sample solution was incubated with 40 µL streptavidin magnetic beads (HY‐K0208) for 4 h at RT, and beads were washed with 1 mL PBS containing 0.1%SDS (once), and 6 mol L^−1^ urea (thrice) and PBS (twice). For target identification by LC‐MS/MS, the enriched proteins from streptavidin beads were separated by SDS‐PAGE followed by Coomassie staining and silver staining. The differentially expressed protein bands to the specific molecular weight region (45–60 kDa) were excised, cut into small pieces, and then washed with 25 mmol L^−1^ ammonium bicarbonate buffer and 50% acetonitrile in 25 mmol L^−1^ ABB buffer. After dehydration in Speedvac, the samples were reduced by dithiothreitol (DTT) and alkylated by iodoacetamide (IAA). Then the samples were incubated with trypsin to digested into peptides overnight at 37 °C. The peptide solution was desalted on the C18 column. Finally, the samples were analyzed by LC‐MS/MS (Thermo Scientific). For pull‐down‐Western blot analysis, the bound proteins were detected by Western blot assay with the same procedures mentioned above.

### RNA‐Sequencing

RAW264.7 cells were seeded in a 12‐well plate at a density of 5 × 10^4^ cells/well overnight, and then incubated with different concentrations of Nef with or without Ox‐LDL (100 µg mL^−1^). After 24 h of treatment, the samples were sent to Shanghai Biotechnology Corporation (Shanghai, China) for the mRNA‐seq analysis (*n* = 3). Total RNA was extracted from RAW264.7 cells followed by library preparation according to Illumina standard instruction (VAHTS Universal V6 RNA‐seq Library Prep Kit for Illumina). Agilent 4200 bioanalyzer was employed to evaluate the concentration and size distribution of the cDNA library before sequencing with an Illumina novaseq6000. The protocol of high‐throughput sequencing was fully according to the manufacturer's instructions (Illumina). The raw reads were filtered by Seqtk before mapping to the genome using Hisat2 (version:2.0.4). The fragments of genes were counted using stringtie (v1.3.3b) followed by TMM (trimmed mean of M values) normalization. Significant differential expressed genes (DEGs) were identified as those with a False Discovery Rate (FDR) value above the threshold (Q < 0.05) and fold‐change >2 using edgeR software. Each group of samples was sequenced with three independent biological replicates. For GO term and KEGG pathway enrichment analyses, the data were analyzed using DAVID (https://david.ncifcrf.gov/). In addition, KEGG pathway maps were acquired from the KEGG database (http://www.kegg.jp/). Finally, several significant genes were also visualized and networks were constructed using the STRING database (http://string‐db.org/) and Cytoscape software (https://cytoscape.org/).

### Quasi‐Targeted Metabolomics Analysis

Lipid and tricarboxylic acid cycle metabolites were determined by quasi‐targeted metabolomics by HPLC‐MS/MS (Novogene, Beijing, China). Experimental methods were performed as described previously.^[^ ^51^ ^]^ Briefly, RAW 264.7 cells (2 × 10^7^) were individually grounded with liquid nitrogen, and the homogenate was resuspended with prechilled 500 µL 80% methanol and 0.1% formic acid by well vortexing. 50 µL of hemolymph samples were mixed with 400 µL prechilled methanol by vortexing. All samples were incubated on ice for 5 min and then centrifuged at 15000 × g, at 4 °C for 10 min. The supernatant was diluted to a final concentration containing 53% methanol by LC‐MS grade water. The samples were then transferred to a fresh vial and centrifuged at 15 000 rpm, 4 °C for 20 min. Finally, the supernatant was injected into the LC‐MS/MS system, and the analyses were performed using an ExionLC AD system (SCIEX) coupled with a QTRAP 6500+ mass spectrometer (SCIEX). Samples were injected onto an Xselect HSS T3 Column (2.5 µm,150 mm × 2.1 mm) using a 30‐min linear gradient at a flow rate of 0.4 mL min^−1^ for the positive polarity mode. Eluent A was 0.1% formic acid‐water, and eluent B was 0.1% formic acid‐acetonitrile. The solvent gradient was set as follows: 2% B, 2 min; 100% B, 15.0 min; 100% B, 17.0 min; 98% B, 17.1 min; 2% B, 20 min. QTRAP 6500+ mass spectrometer was operated in positive polarity mode with curtain gas of 35 psi, collision gas of medium, ion spray voltage of 5500 V, the temperature of 550 °C, ion source gas of 1:60, and ion source gas of 2:60. For negative ion mode, The solvent gradient was set as in the positive ion mode. QTRAP 6500+ mass spectrometer was operated in negative polarity mode with curtain gas of 35 psi, collision gas of medium, ion spray voltage of −4500 V, the temperature of 550 °C, ion source gas of 1:60, and ion source gas of 2:60. Detection of the experimental samples using MRM was based on Novogene in‐house database. Q3 (daughter) was used for the quantification. Q1 (parent ion), Q3, retention time, declustering potential, and collision energy were used for metabolite identification. Data files generated by HPLC‐MS/MS were processed with SCIEX OS (version 1.4) to integrate and correct the peaks. A total of 326 compounds were identified in the RAW264.7 cell. Metabolomics data analysis was then performed using MetaboAnalyst 4.0.

### Cellular Thermal Shift Assay (CETSA)‐Western Blot (WB)

The CETSA‐WB experiment was carried out as previously described.^[^ ^52^ ^]^ Briefly, the soluble protein lysate of RAW264.7 cells was aliquoted into PCR tubes and treated with Nef (200 µm) or DMSO for 1 h at RT prior to CETSA heat pulse. The solutions were heated at the indicated temperatures (42‐67 °C) for 5 min, followed by cooling at 4 °C for 3 min in a thermocycler. After centrifugation for 10 min (12 000 r min^−1^, 4 °C), the soluble supernatant was subject to Western blotting.

### Drug Affinity Responsive Target Stability (DARTS)‐Western Blot (WB)

Cells at 70–80% confluence were cultured in 100 cm^2^ culture plates. After washing once with ice‐cold PBS, the cells were treated with ice‐cold M‐PER lysis buffer (Thermo Fisher Scientific Inc.) supplemented with PMSF (1 mM) and protein phosphatase inhibitors (1 mM). After collecting the cells with a scraper, the cells were incubated for 10 min at 4 °C. The cell lysates were centrifuged at 13 000 rpm for 10 min and the supernatant was diluted with M‐PER lysis buffer to a final protein concentration of 5.8 mg mL^−1^. The protein lysates were mixed with 10 × TNC buffer (500 mm Tris‐HCl, pH 8.0, 500 mM NaCl, and 100 mM CaCl_2_). The lysates in 1× TNC buffer were split into 1.5 mL tubes and incubated with DMSO or Nef (2.5, 5, 10 µm) for 1 h at room temperature. Following the incubation, each sample was split into 50 µL of aliquots (50 µg in proteins) and proteolyzed in various concentrations of pronase (Sigma, 10165921001) for 10 min at room temperature. After 10 min, digestion was further stopped by adding 5 × sample loading buffering and boiling at 100 °C for 5 min. An equal portion of each sample was then loaded onto 10% SDS‐PAGE gels for Western blotting.

### Recombinant Human eEF1A1 Proteins Expression and Purification

The recombinant human eEF1A1 proteins were expressed and purified as described previously.^[^ ^53^, ^54^ ^]^ Briefly. PET‐28A‐eEF1A1 plasmid was transformed into *Escherichia coli* (BL21 (DE3)), After the bacterial growth in a bacteria culture medium containing 1% ampicillin at 37 °C, 190 r min^−1^ overnight. Then, bacteria were removed from the thermostatic shaking bed and equilibrated to room temperature, induction was carried out at 18 °C using 0.5 mM isopropyl‐β‐D‐thiogalactoside (IPTG), and growth was continued at 18 °C for ≈8 h. The bacteria were collected by centrifugation, and the obtained pellet was either stored at −80 °C or used freshly for the subsequent steps. The pellet was resuspended in lysis buffer A (250 mM NaCl, 40 mM Tris, pH 8.0, and 10 mM imidazole) containing protease inhibitor PMSF (1 mm) (solabio, China) and 2 mM β‐mercaptoethanol (β‐ME) (final), the bacteria were lysed by microfluidizer, and bacteria debris was removed by ultracentrifugation (14 000 r min^−1^, 1 h, 4 °C). Then, the Ni‐agarose beads were rinsed with lysis buffer B (500 mm NaCl, 20 mMm Tris, and 250 mM imidazole) and equilibrated three times with lysis buffer A, and the supernatant was incubated for 1 h with Ni‐agarose beads, the loaded beads were then washed with lysis buffer containing 2 mm β‐ME and protease inhibitor PMSF (1 mm), and the protein was eluted with buffer A and buffer B. The purity of the obtained proteins was confirmed by Coomassie brilliant blue staining and Western Blot.

### Isothermal Titration Calorimetry (ITC)

Isothermal titration calorimetry (MicroCal PEAQ‐ITC Analysis, USA) was employed to confirm that eEF1A1 was the direct target of Nef, which was a highly sensitive analytical technique traditionally used to provide a complete thermodynamic profile for molecule‐protein interactions to elucidate the assembly of cellular components, regulation of enzymatic activities, and signal transduction, to directly quantify the binding association constants (Ka), enthalpy changes (ΔH), entropy changes (ΔS), and Gibbs free energy changes (ΔG)^[^ ^55^ ^]^ of the system to examine the influence of Nef and eEF1A1 binding interactions.

### Plasmid and siRNA Transfection

pcDNA‐eEF1A1, EGFP‐ARID3A, mCherry‐eEF1A1, pcDNA‐ARID3A, pcDNA‐ARID3A‐461A, pcDNA‐ARID3A‐491, pcDNA‐vectors, mENO2 promoter pGL3‐Basic, mENO2 promoter MUT1 pGL3‐Basic and mENO2 promoter MUT2 pGL3‐Basic were designed and synthesized by Youbao Bio (Chang sha, China). siRNA‐eEF1A1 target sequence(5^,^ ‐AGAAGAGATACGAGGAAAT‐3^,^)was purchased from Ruibio (Guangzhou, China), and siRNA‐ARID3A(Forward:5^,^ ‐GCAUGUCGGUGGAGAUCAACGTT‐3^,^; Reverse:5^,^ ‐CGUUGAUCUCCACCGACAUGCTT‐3^,^)was purchased from Gen CREATE (Changsha, China). Plasmid extraction was performed according to the instructions of the plasmid extraction kit (TianGen, Beijing, China). These pcDNA‐eEF1A1 or pcDNA‐vectors were transfected into RAW264.7 cells or 293T cells by ExFect transfection reagent (Vazyme, Nanjing, China). siRNA‐eEF1A1 and siRNA‐ARID3A were transfected into RAW264.7 cells by RNAfit (HANHEN, Shanghai, China), According to the instructions of the kit. After transfecting the cells for 24 h, they were utilized for subsequent drug administration treatments.

### Chromatin Immunoprecipitation (ChIP)‐qPCR

293T cells and RAW264.7 cells were seeded on 10 cm^2^ culture dishes and divided into Control, Model, and Nef (5 µm) groups with or without pcDNA‐ARID3A and siRNA‐ARID3A. Cell samples were collected 24 h after treatment, and ChIP experiments were performed as described in the kit instructions (BeyoChIP Enzymatic ChIP Assay Kit, China, P2083S) and previous literature.^[^ ^56^ ^]^ Briefly, crosslinking was performed with 1% formalin, and the cells were lysed in SDS buffer and sonication was used to fragment the DNA. Eluted DNA fragments were analyzed by qPCR, as described in ″Quantitative Real‐Time Polymerase Chain Reaction″. The primers are listed in the section “ChIP‐qPCR” in (Table S2, Supporting Information).

### Measurement of Mitochondrial Function and ECAR

For Seahorse analysis, RAW264.7 or 293T cells were seeded overnight in quadruplicate at a density of 1 × 10^4^ cells per well on a pretreated XFe 24‐well Seahorse microplate in DMEM medium with 10% FBS. Cells were treated and transfected as described in the previous methods. Prior to starting the OCR assay, cells were washed and incubated in XF Base Medium supplemented with 10 mm glucose, 1 mm sodium pyruvate, and 2 mm L‐glutamine in 37 °C incubator without CO_2_ for 1h. Oligomycin (ATPase inhibitor, 1.5 µm), FCCP (1 µm), and Rotenone/Antimycin A (0.5 µm /0.5 µm) were injected where indicated and the OCR (pMoles O_2_/min) was measured in real‐time. Prior to starting the ECAR assay, cells were washed and incubated in XF Base Medium supplemented with 2 mm L‐glutamine in 37 °C incubator without CO_2_ for 1h. Oligomycin (1 µm), 2‐DG (50 mm), and glucose (10 mm) were injected where indicated and the ECAR (mpH min^−1^) was measured in real time. Proteins from each experimental group were extracted for normalization. Data were acquired with an Aurora flow cytometer (Cytek, California) and analyzed with GraphPad Prism 8.0 software.

### Luciferase Reporter Assay

Luciferase reporter assay was performed according to a standard protocol as described previously.^[^ ^57^ ^]^ Briefly, 293T cells (3 × 10^4^ cells/well) were seeded in 24‐well plates in triplicate and allowed to settle for 24 h. The indicated plasmids and 0.5 ng pRL‐TK Renilla plasmid were transfected by ExFect transfection reagent (Vazyme, Nanjing, China), and plasmids were designed and synthesized by Youbao Bio (Chang sha, China). At 24 h post‐transfection, luciferase and Renilla signals were determined by a Dual Luciferase Reporter Assay Kit (YESEN, China, Cat. 11401ES60) according to the manufacturer's instructions.

### Immunofluorescence Assay

Paraffin slices were washed with phosphate‐buffered saline (PBS) three times, and incubated with 0.1 mL Triton X‐100 (0.5%) for 20 min and blocked for 20 min with 5% bovine serum albumin (BSA, A8020, Solaibio, China). Then, the slices were incubated with primary antibodies (anti‐CD86 and anti‐CD206, GB115630/GB113497, servicebio, Wuhan, China) at 4 °C for 8–10 h, washed with PBS, and incubated with fluoresceinconjugated goat anti‐rabbit secondary antibodies (GB23204, servicebio, Wuhan, China) for 1 h. Then, different fluorescence was labeled with the corresponding TSA dye. Nuclei were stained using 4,6‐diamidino‐2‐phenylindole (DAPI, GeneCopoeia) for 20 min, and washed with PBS 3 times. The stained photographs were acquired from a fluorescence microscope (SLIDEVIEW VS200, OLYMPUS), and analysed by ImageJ software. *p* < 0.05 was considered statistically significant.

### Computational Method

The protein sequence of known eEF1A1 and ARID3A was downloaded from NCBI for AlphaFold2 modeling.^[^ ^58^ ^]^ The obtained protein structure of eEF1A1 was used to perform pocket searching with AutoDock Vina,^[^ ^59^ ^]^ and molecular docking was performed using neferine as a ligand. For eEF1A1 and ARID3A protein‐protein interactions, H‐DOCK^[^ ^60^ ^]^ was used to predict the protein‐protein binding complex. Molecular dynamics (MD) simulations were performed for both ligand‐protein and protein‐protein complex structures to evaluate the binding pose, and the most suitable ligand‐protein and protein‐protein structures were used for further analysis. The MD simulations were carried out using AMBER 20. The protein force field ff14SB,^[^ ^61^ ^]^ the gaff force field for small molecules, and the TIP3P^[^ ^62^ ^]^ model for water molecules were employed. The RESP charges of ligands were calculated using Gaussian (HF/6‐31G^*^). The initial coordinate and topology files were generated by using the tleap program.^[^ ^63^ ^]^ Both protein‐protein and protein−ligand complexes were subjected to 50 ns molecular dynamics simulations, with all simulations performed in triplicate for enhanced reliability. The equilibrated trajectory files were analyzed using cpptraj.^[^ ^64^ ^]^ The binding free energy of the protein−ligand complexes was calculated using MMGBSA.^[^ ^65^ ^]^

### Statistical Analysis

All data are presented as mean ± standard error of the mean (SEM) for 3 independent experiments. All statistical analyses were carried out using SPSS version 21.0 statistical software (SPSS Inc., New York, NY, USA). The two‐tailed paired Student's *t*‐test was used to assess the comparisons between groups. Other statistical analyses were carried out by one‐way ANOVA followed by Tukey's test in multiple groups, *p* < 0.05 was considered statistically significant. Statistical graphs were performed in GraphPad Prism 8.0 software (San Diego, CA, USA).

## Conflict of Interest

The authors declare no conflict of interest.

## Author Contributions

B.X. and L.‐W.T. contributed equally to this work. B.X. conducted the experiments, analyzed the data, and wrote the manuscript; L.‐W.T. designed and synthesized probes and wrote the manuscript; C.L. conducted the computational analysis; J.L. and X.T. conducted the animal experiments; R.Z. provided the resources and edited the manuscript; F.Z. conducted the computational analysis and edited the manuscript; Z.L. and Y.C. supervised the project and edited the manuscript.

## Supporting information



Supporting Information

## Data Availability

The data that support the findings of this study are available from the corresponding author upon reasonable request.
