# Supplementary material for: Disruption of the eEF1A1/ARID3A/PKC‐δ Complex by Neferine Inhibits Macrophage Glycolytic Reprogramming in Atherosclerosis
Source: Adv Sci (Weinh). 2025 Feb 20;12(15):2416158. doi: 10.1002/advs.202416158 (PMC12005739; doi:10.1002/advs.202416158)
Supplement: Supplementary file 1 — Supporting Information [file ADVS-12-2416158-s001.docx]

**SUPPLEMENTARY DATAS**


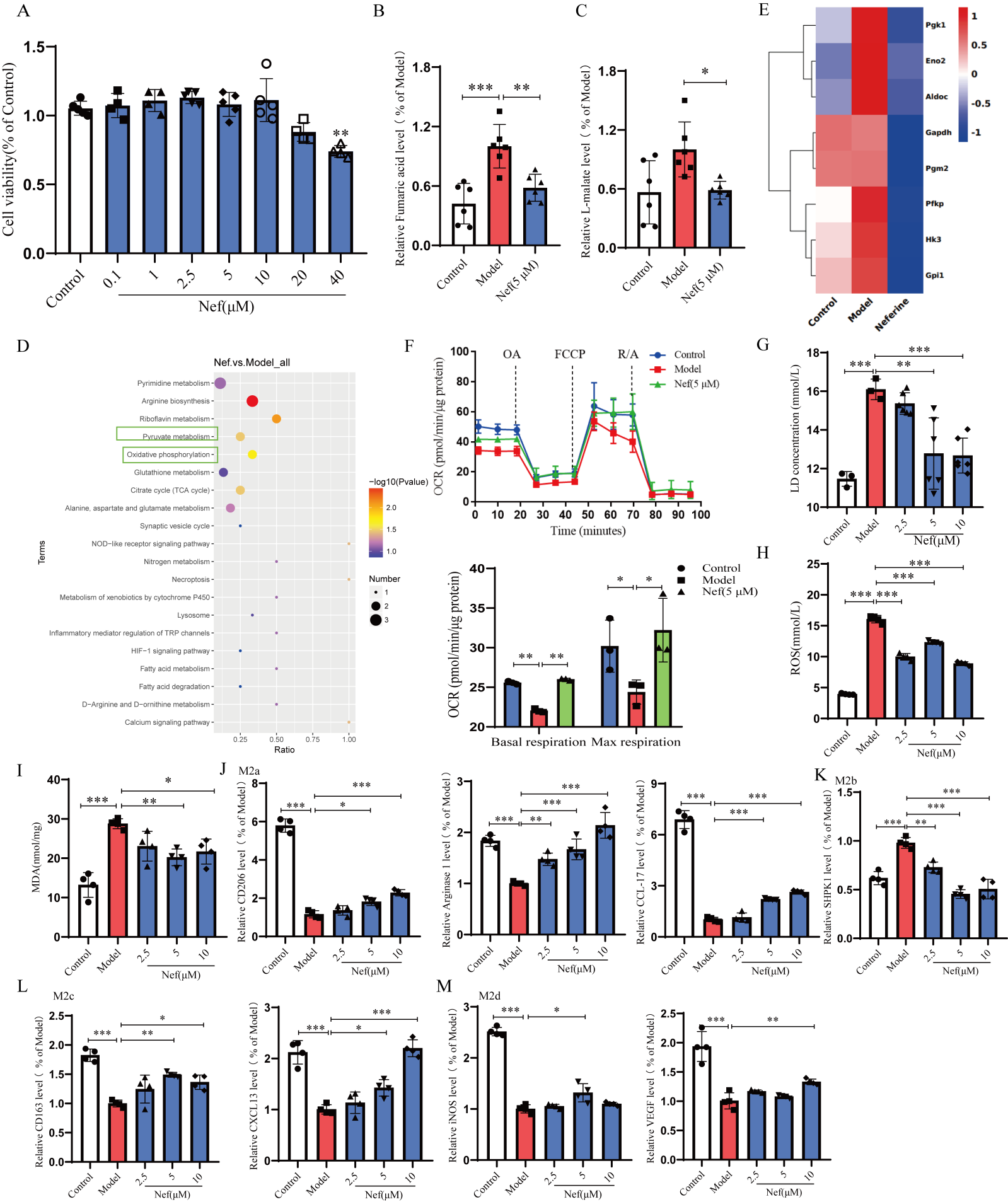


**Figure S1. Nef inhibited ox-LDL-induced glycolytic reprogramming without cytotoxicity.** (A) Effect of Nef on the cytotoxicity of RAW264.7 cells.(B) Effect of Ox-LDL on the proliferation of RAW264.7 cells; (C and D) Nef inhibited the accumulation of fumarate and L-malate; (E) KEGG analysis was used to analyze the effect of Nef on ox-LDL-induced macrophage metabolism; (F) Nef promoted OXPHOS in OX-LDL-treated macrophages; (G-I) Nef inhibited ox-LDL-induced the increase of extracellular lactate, MDA and ROS levels. (J-M) The effect of Nef on the expression of biomarkers for each subtype of M2 macrophages; **P*< 0.05，***P*< 0.01, vs Model group, (n = 4).


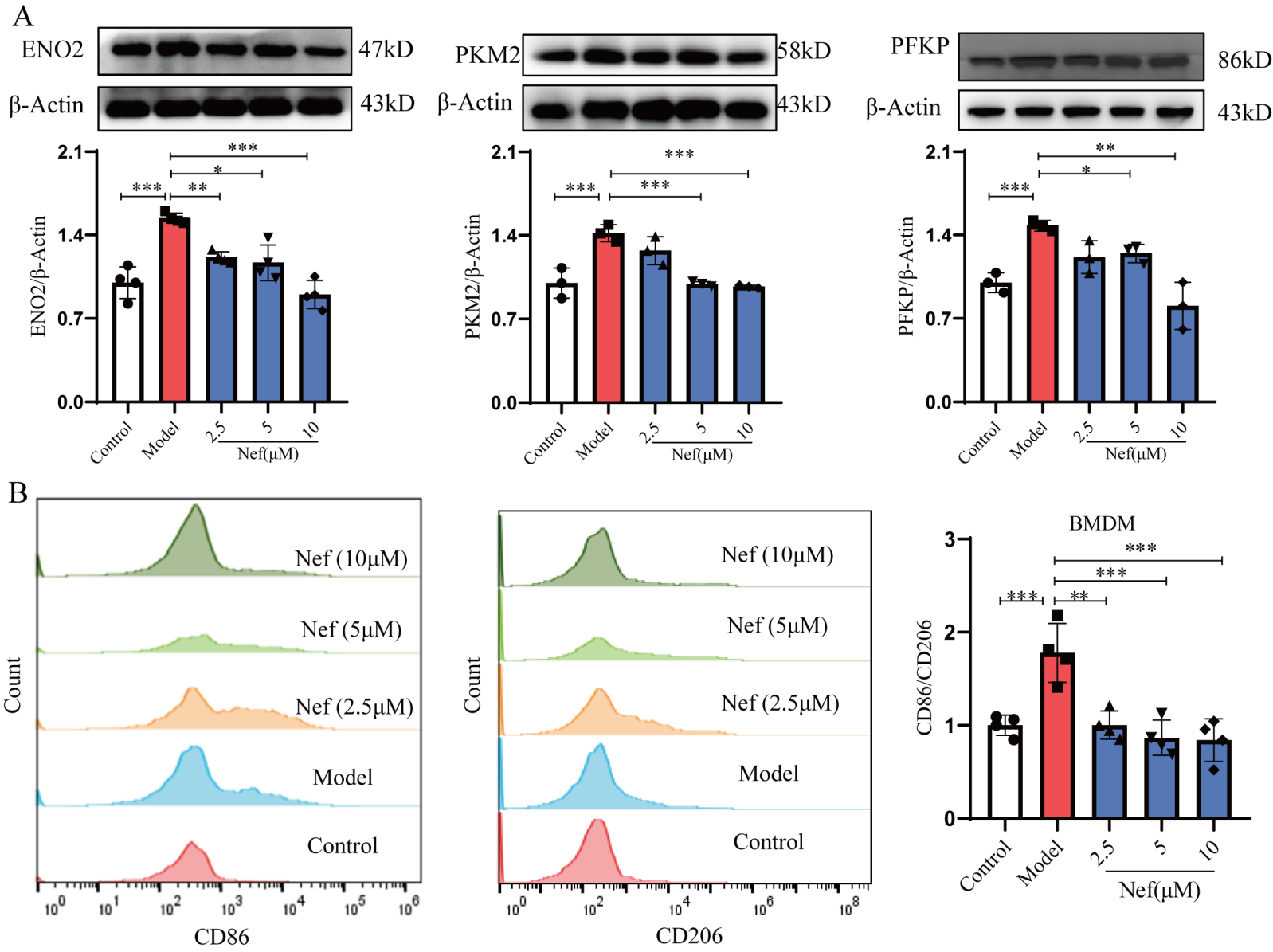


**Figure S2. Nef inhibited ox-LDL-induced glycolytic proteins and promoted macrophage polarization from M1 to M2 on primary macrophages.** (A) Effect of Nef on the expressions of glycolytic related proteins. (B) Flow cytometry analysis for the ratio of CD86/CD206. **P*< 0.05，***P*< 0.01, ****P*< 0.001 vs Model group, (n = 3~4).


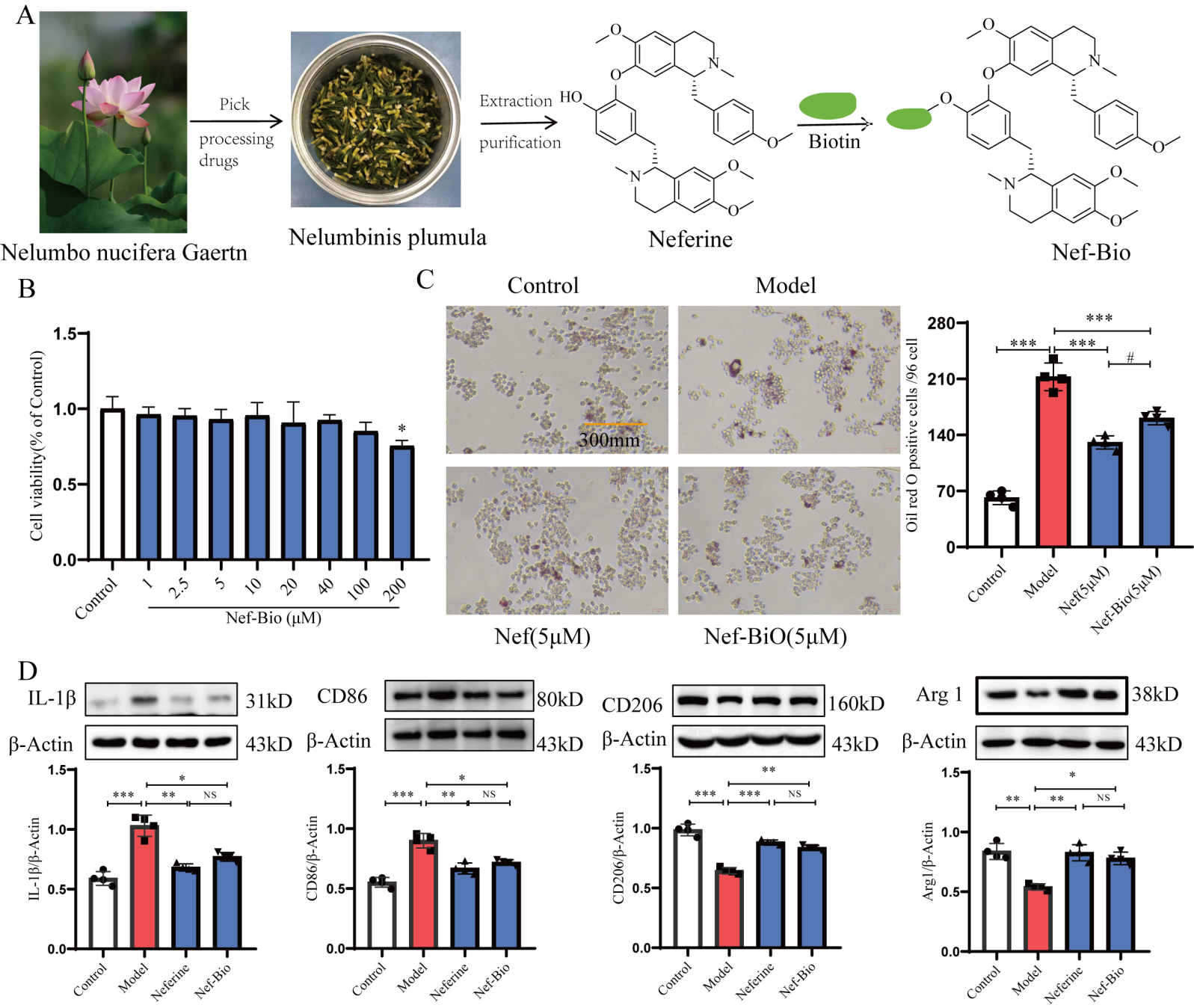


**Figure S3. Synthesis and pharmacodynamic validation of a biotin-coupled neferine probe.** (A) Process of Nef-Bio probe synthesis. (B)Effect of Nef-Bio on macrophage toxicity. (C) Nef-Bio inhibited macrophage foam cell formation. (D) Nef-Bio inhibited ox-LDL-induced M1 macrophage polarization and promote M2 macrophage polarization. **P*< 0.05，***P*< 0.01, vs Model group. **P*< 0.05，vs Nef (5 μM), scale column:100 μm, (n = 4).


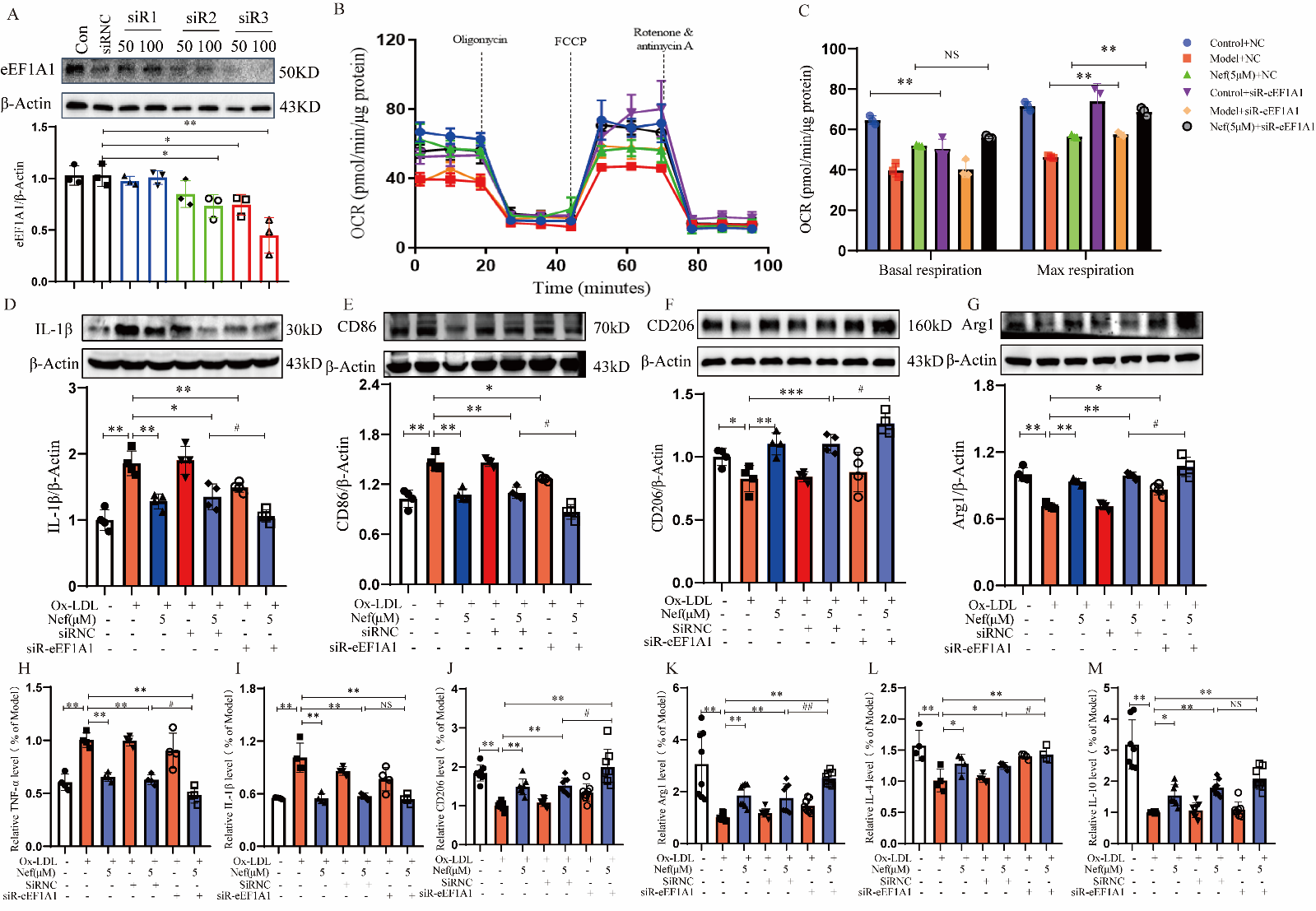


**Figure S4. Neferine inhibited ox-LDL-induced M1 macrophage polarization and inflammatory cytokine secretion by targeting eEF1A1.** (A) siRNA targeting eEF1A1 was screened. (B and C) Effect of Nef on OXPHOS in ox-LDL-induced macrophages after silencing eEF1A1. (D and E) Effect of Nef on ox-LDL-induced macrophage polarization after silencing eEF1A1. (H-M) Effect of Nef on the mRNA levels of biomarkers for M1 or M2 macrophage after silencing eEF1A1.**P*< 0.05，***P*< 0.01, vs Model group. ^#^*P*< 0.05, vs Nef (5μM) +siRNC group, (n = 3)


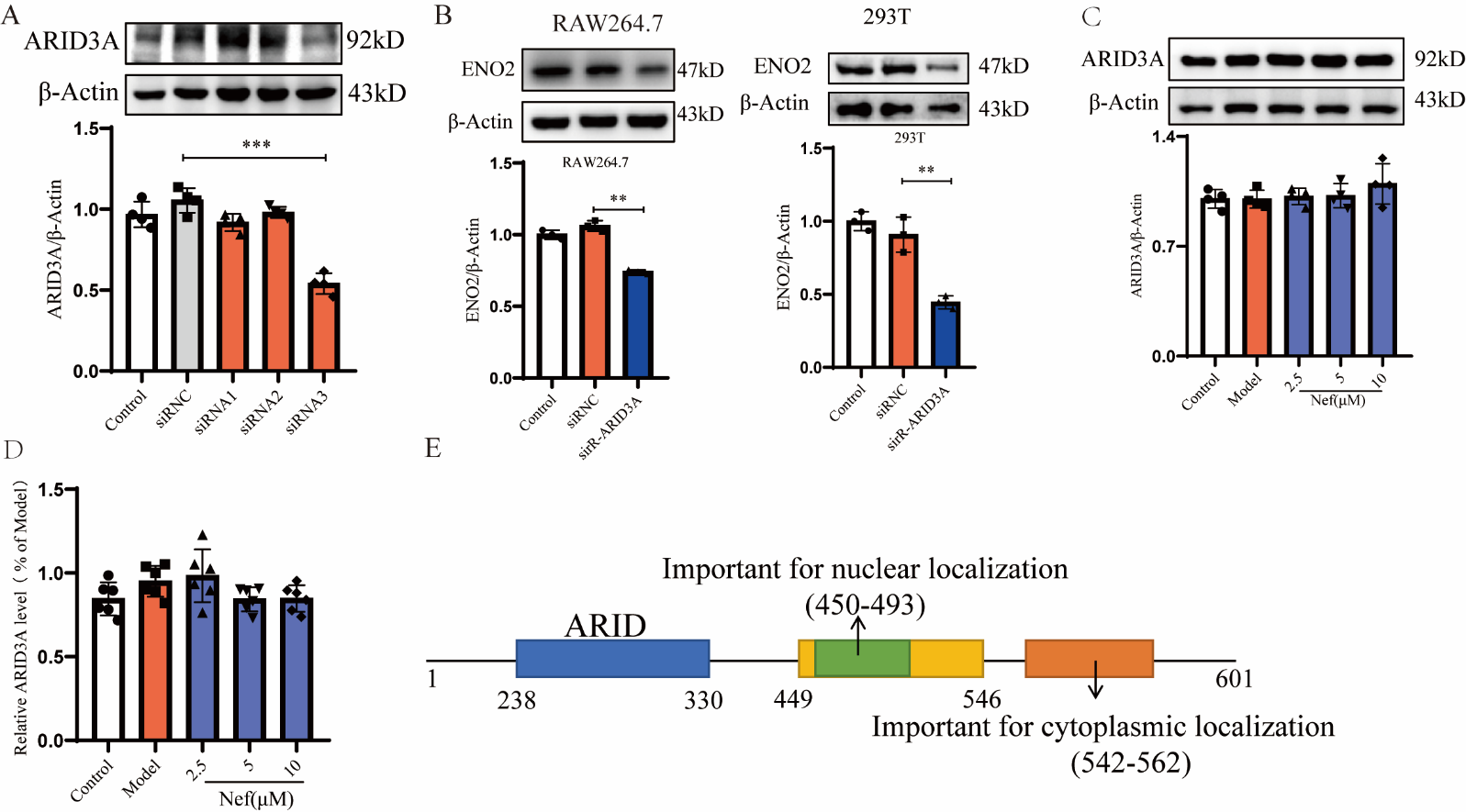


**Figure S5. Effect of neferine on ARID3A expression.** (A) siRNA targeting ARID3A was screened. (B) Silencing ARID3A significantly inhibited the expression of ENO2 in RAW264.7 and 293T cells. (C and D) Nef did not affect the expression of ARID3A protein and mRNA induced by Ox-LDL. (E) Structural and functional analysis of ARID3A. **P*< 0.05，***P*< 0.01, vs siRNC group, (n = 4).


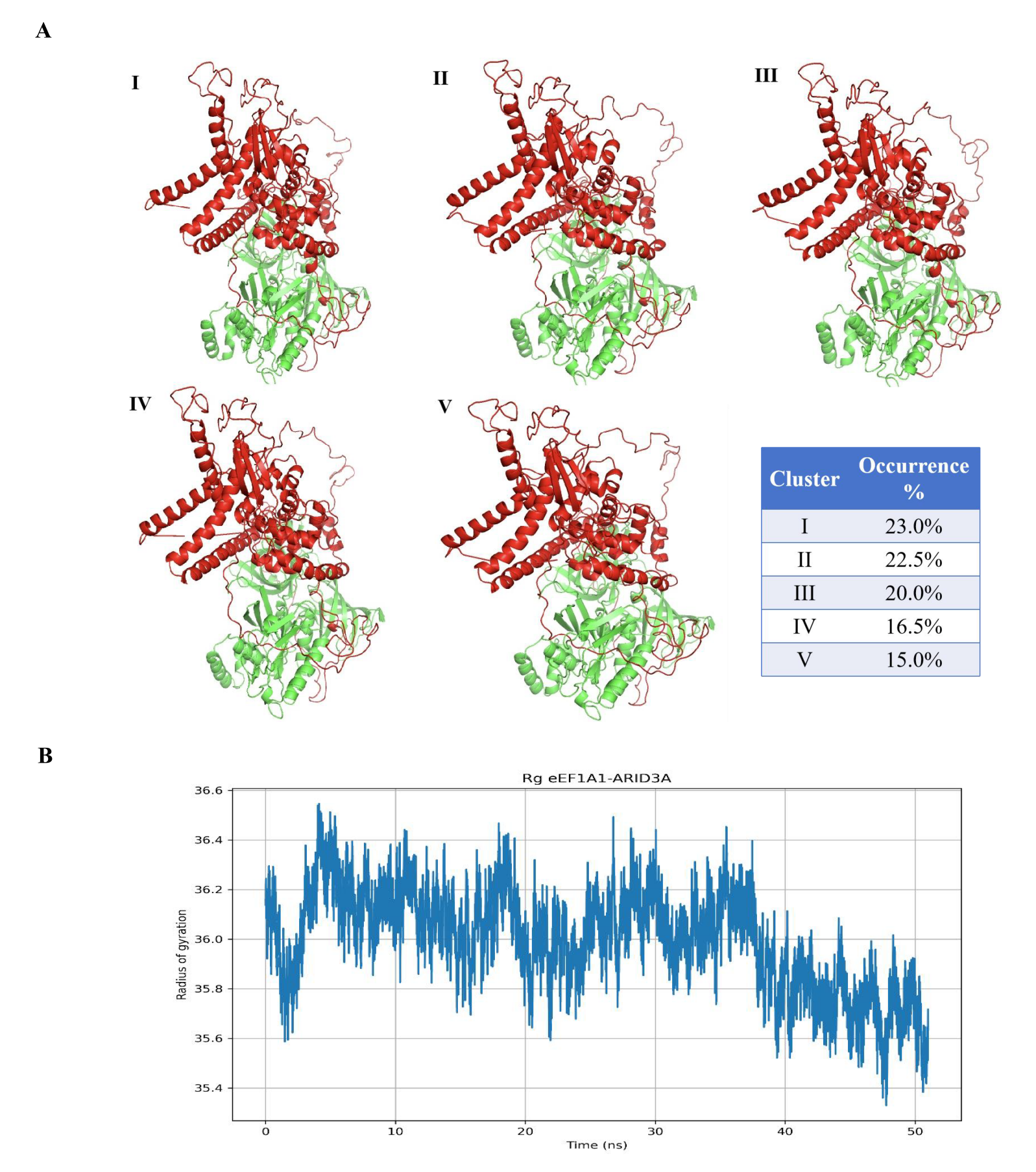


**Figure S6.** (A) Cluster analysis of eEF1A1-ARID3A, five major cluster structures are labeled as I-V. The cluster occurrence percentage is shown respectively. The eEF1A1 and ARID3A are rendered in green and red respectively. (B) Radius of gyration (Rg) analysis of eEF1A1-ARID3A.

**Figure S7. The eEF1A1-neferine binding structures of molecular docking.** (A-F) Six ligand-protein binding complex structures were predicted by pocket searching and ligand docking. The structures are arranged in order from A to F according to better calculated binding energy.

Table S1. qPCR primer sequences

| Name | Forward | Reverse |
| --- | --- | --- |
| Arg1 | CGGCAGTGGCTTTAACCTTG | TTGGGAGGAGAAGGCGTTTG |
| CD206 | TAGCACTGGGTTGCATTGGT | TGCAGGGTTGACATGAGACC |
| CCL17 | ACCGCTCATCTGTGCAGACC | CGCCTGTAGTGCATAAGAGTCC |
| CD150 | GAGAACGTTTCTGTTCAGCAAT | CGTTCTCCTGGGTTTTGTTTAG |
| SPHK1 | CTTCTCATTGGACTGTGGTACC | CGTAGAACAGATGCATAACACC |
| CD163 | GTTTGTGGAGCCATTCTATTGG | GGAAACTGTAAGTCGCTGAATC |
| CXCL13 | GGCCACGGTATTCTGGAAGC | ACCGACAACAGTTGAAATCACTC |
| VEGF | TAGAGTACATCTTCAAGCCGTC | CTTTCTTTGGTCTGCATTCACA |
| iNOS | AAAGTGACCTGAAAGAGGAAAAGGA | TTGGTGACTCTTAGGGTCATCTTGTA |
| β-Actin | TGACGTTGACATCCGTAAAGACC | GCTAGGAGCCAGAGCAGTAATC |
| IL-1β | TGGCAACTGTTCCTG | GGAAGCAGCCCTTCATCTTT |
| TNFa | GCCTCTTCTCATTCCTGCTT | TGGGAACTTCTCATCCCTTTG |
| IL-10 | AGGCGCTGTCATCGATTT | CACCTTGGTCTTGGAGCTTAT |
| IL-4 | GGTCTCAACCCCCAGCTAGT | GCCGATGATCTCTCTCAAGTGAT |
| Glut1 | AGAAGAGGGTCGGCAGATGATG | TGAGTAGTAGAACACAGCATTGATACC |
| PFKP | AAAGAAGATTTAAGGAAGCCGTGAAAC | ATGGCAAGACGCTTATAGGTGTTC |
| ENO2 | AGATAGTGGGCGATGACCTGAC | GCTTGGATGGCTTCTGTGACC |
| HIF1α | AGCAATTCTCCAAGCCCTCCAAG | GATTCATCAGTGGTGGCAGTTGTG |

Table S2. sequencing of ChIP

| Name | Forward | Reverse |
| --- | --- | --- |
| ARID3A(h-ENO2) | TTGCGAGGTAGTCTTGCTTAGTCTC | GGAGGCTGAGGCAGGAGAATTG |
| ARID3A(m-ENO2) | GACACGCAGGAGACCACAGTAG | GCTACATCCTCCACGCACCAC |
